# Supplementary material for: High performance TadA-8e derived cytosine and dual base editors with undetectable off-target effects in plants
Source: Nat Commun. 2024 Jun 14;15:5103. doi: 10.1038/s41467-024-49473-w (PMC11178825; doi:10.1038/s41467-024-49473-w)
Supplement: Supplementary file 1 — Supplementary Information [file 41467_2024_49473_MOESM1_ESM.pdf]

High performance TadA-8e derived cytosine and dual base editors with undetectable off-target effects in plants

Tingting Fan[1,2,7], Yanhao Cheng[3,7], Yuechao Wu[4,5,7], Shishi Liu[1,7], Xu Tang[2,7], Yao He[1], Shanyue Liao[1], Xuelian Zheng[2], Tao Zhang[4,5*], Yiping Qi[3,6*] & Yong Zhang[2*]

[1]Department of Biotechnology, School of Life Sciences and Technology, Center for Informational Biology, University of Electronic Science and Technology of China, Chengdu 610054, China. [2]Chongqing Key Laboratory of Plant Resource Conservation and Germplasm Innovation, Integrative Science Center of Germplasm Creation in Western China (Chongqing) Science City, School of Life Sciences, Southwest University, Chongqing 400715, China. [3]Department of Plant Science and Landscape Architecture, University of Maryland, College Park, Maryland 20742, USA. [4]Jiangsu Key Laboratory of Crop Genetics and Physiology, Co-Innovation Center for Modern Production Technology of Grain Crops, Key Laboratory of Plant Functional Genomics of the Ministry of Education, Yangzhou University, Yangzhou 225009, China. [5]Joint International Research Laboratory of Agriculture and Agri-Product Safety of Ministry of Education of China, Yangzhou University, Yangzhou 225009, China. [6]Institute for Bioscience and Biotechnology Research, University of Maryland, Rockville, Maryland 20850, USA. [7]These authors contributed equally: Tingting Fan, Yanhao Cheng, Yuechao Wu, Shishi Liu, Xu Tang. [*]e-mail: zhangtao@yzu.edu.cn; Yiping@umd.edu; zhangyong916@swu.edu.cn

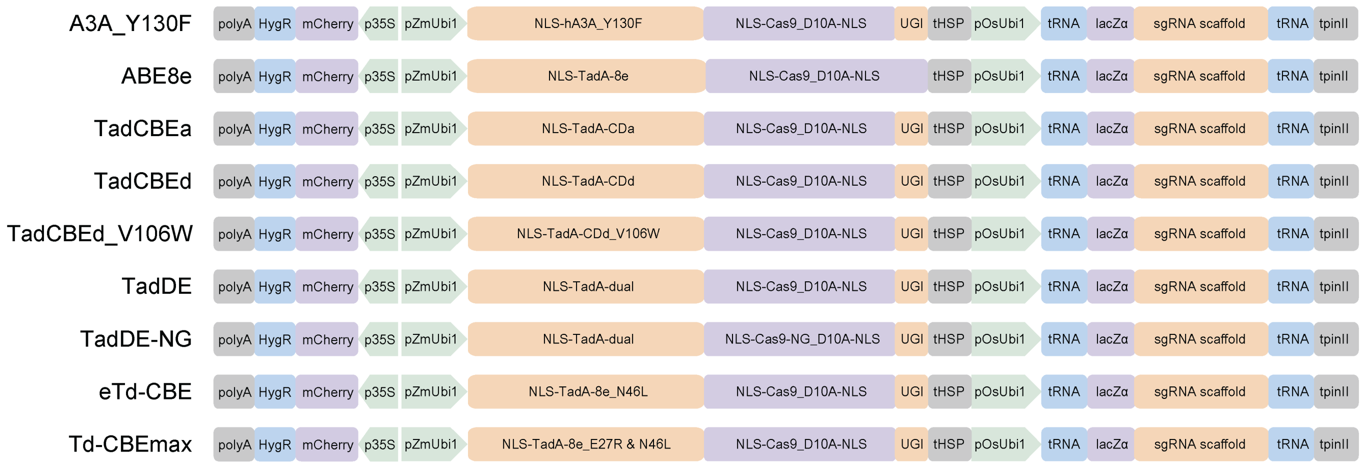

Supplementary Figure 1. Schematic diagrams depicting the detailed construction information of the T-DNA region for base editors evaluated in this study. The deaminases and nCas9 proteins are driven by the *ZmUbi1* promoter, and the sgRNA expression cassette is initiated by the *OsUbi1* promoter.

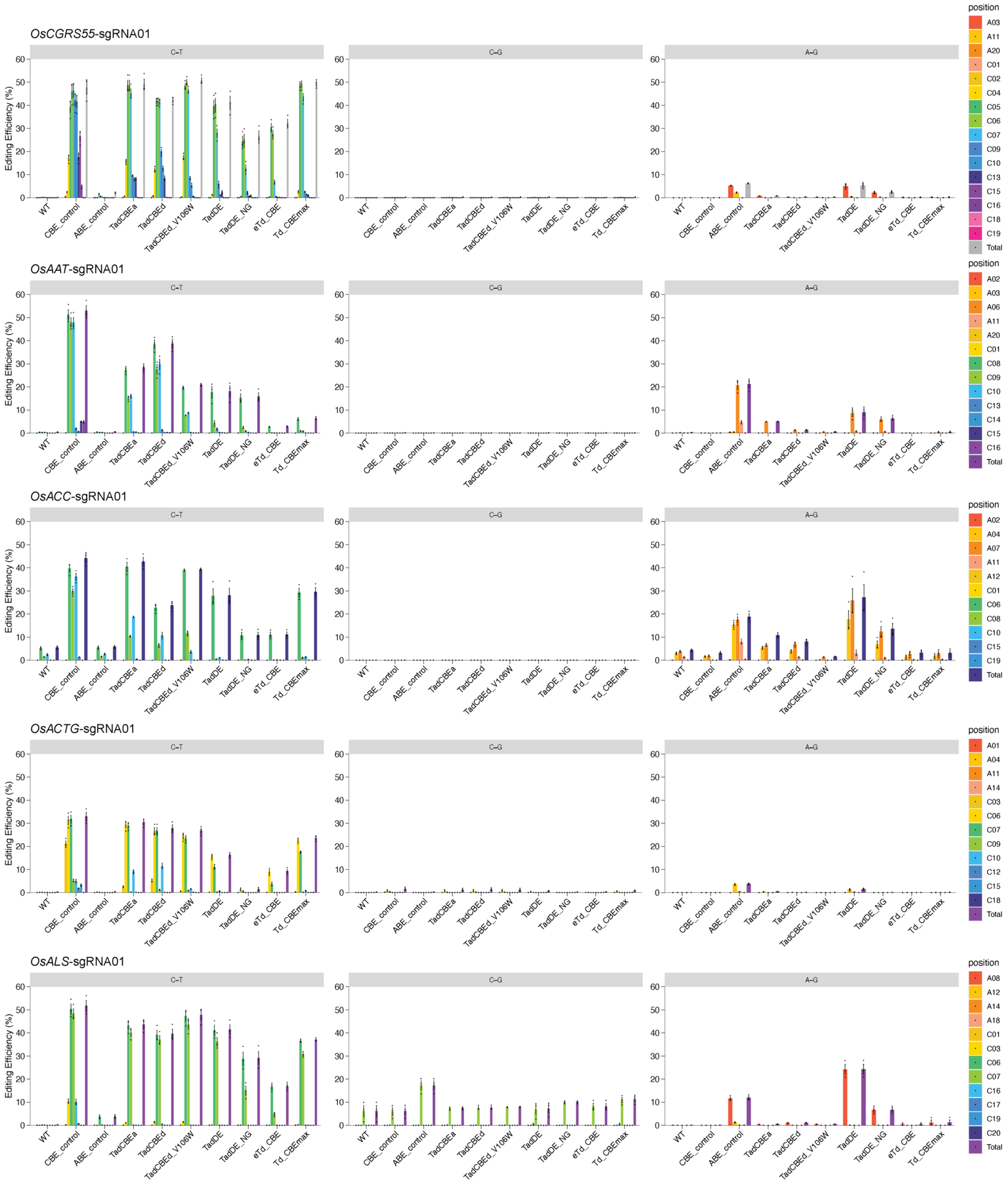

Supplementary Figure 2. Base editing efficiency at multiplex array 01 target sites in rice protoplasts (part 1). Bars of different colors represent the A and C at various positions within the target site. The editing efficiencies of C-to-T and A-to-G at different positions of gRNA array 01 are presented.

(continued)

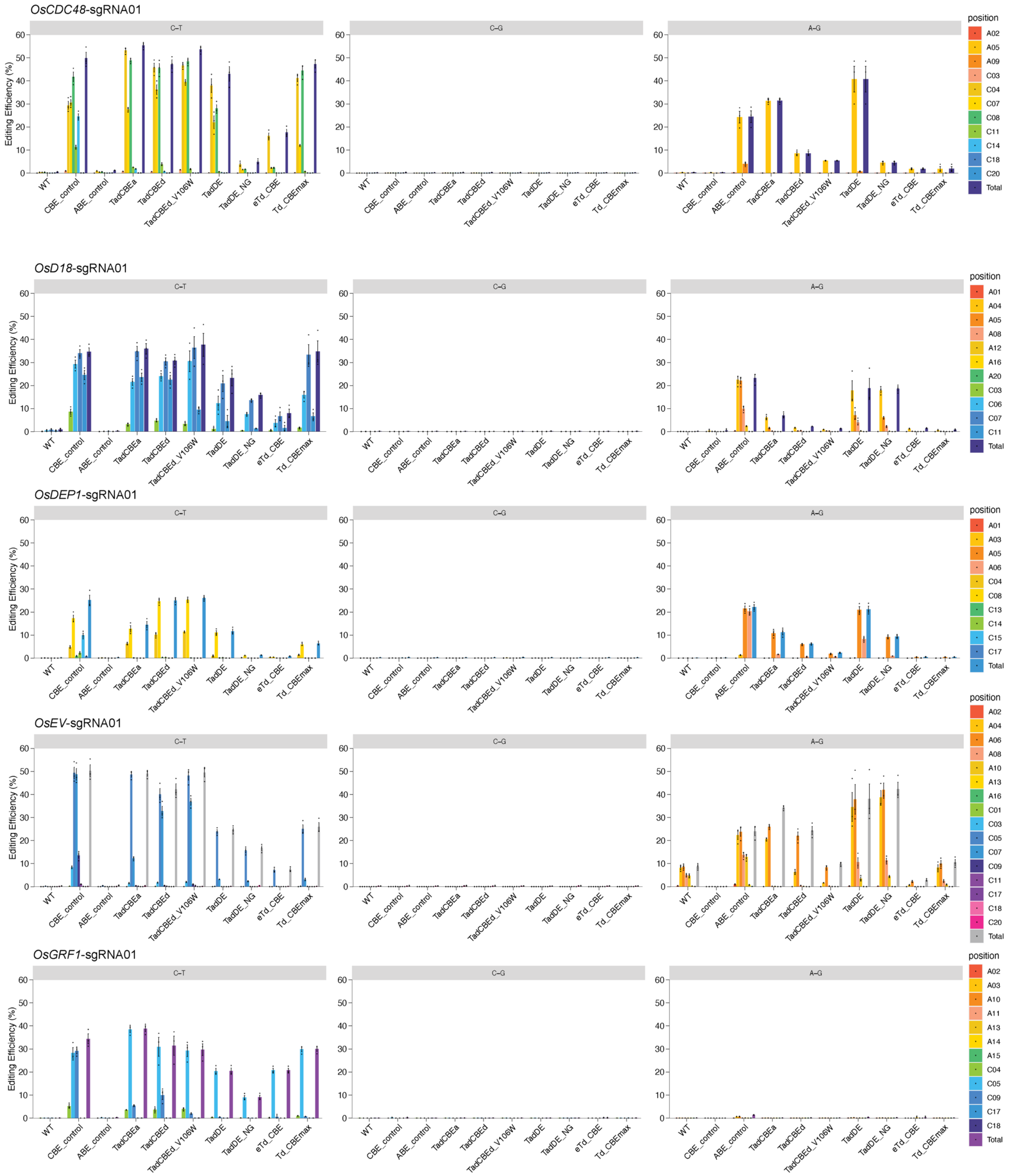

Supplementary Figure 2. Base editing efficiency at multiplex array 01 target sites in rice protoplasts (part 2). Bars of different colors represent the A and C at various positions within the target site. The editing efficiencies of C-to-T and A-to-G at different positions of gRNA array 01 are presented.

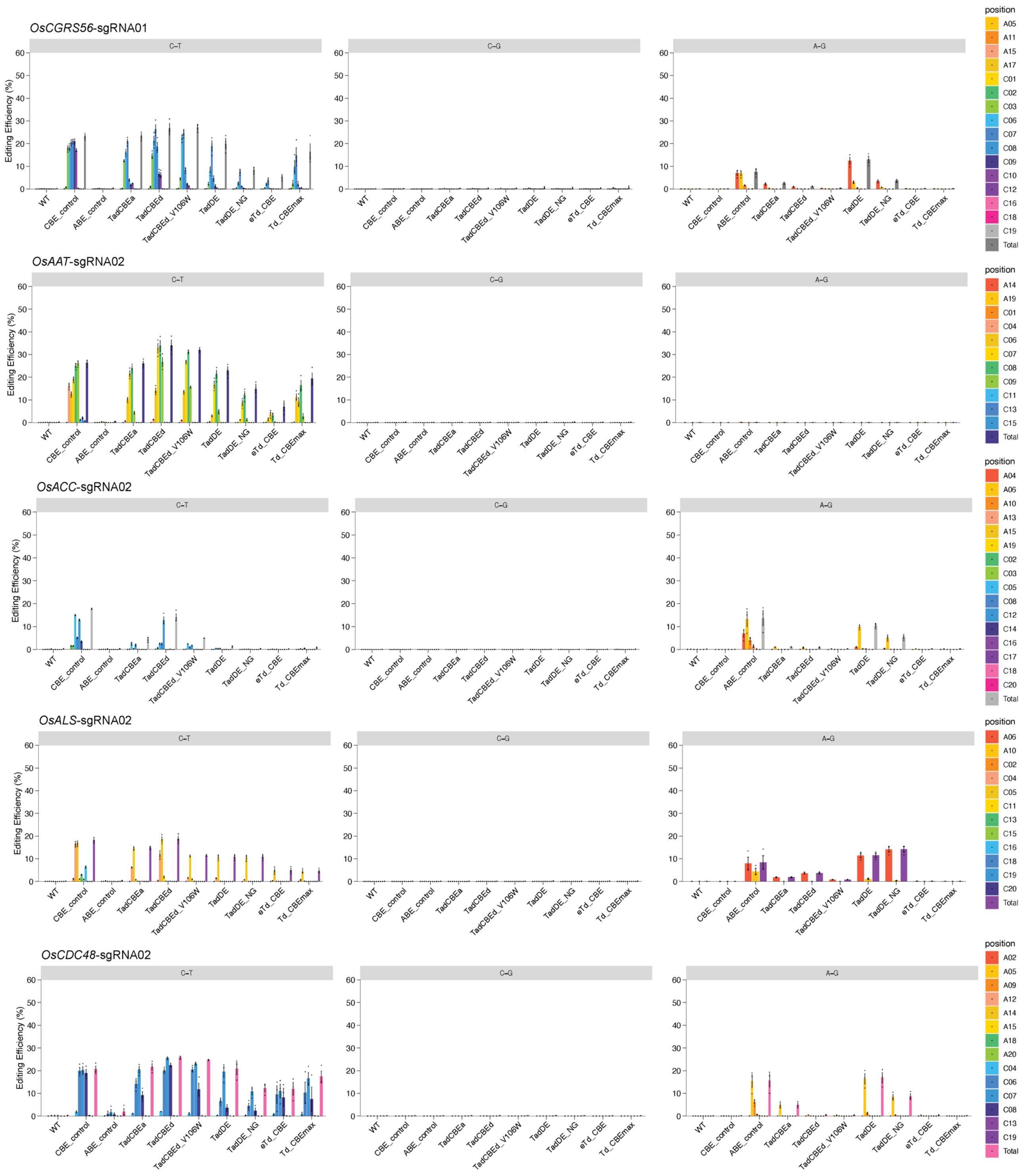

Supplementary Figure 3. Base editing efficiency at multiplex array 02 target sites in rice protoplasts (part 1). Bars of different colors represent the A and C at various positions within the target site. The editing efficiencies of C-to-T and A-to-G at different positions of gRNA array 02 are presented.

(continued)

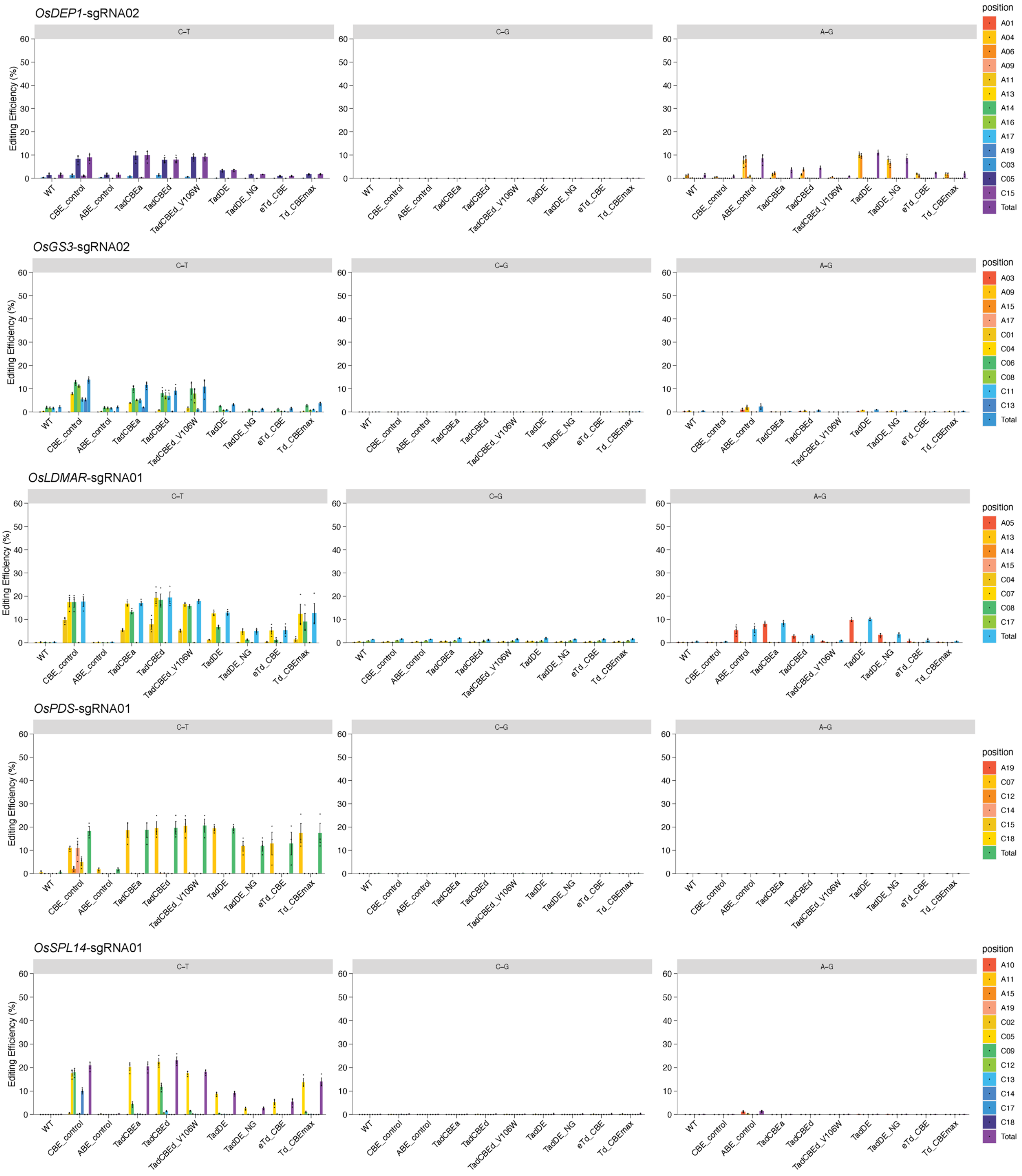

Supplementary Figure 3. Base editing efficiency at multiplex array 02 target sites in rice protoplasts (part 2). Bars of different colors represent the A and C at various positions within the target site. The editing efficiencies of C-to-T and A-to-G at different positions of gRNA array 02 are presented.

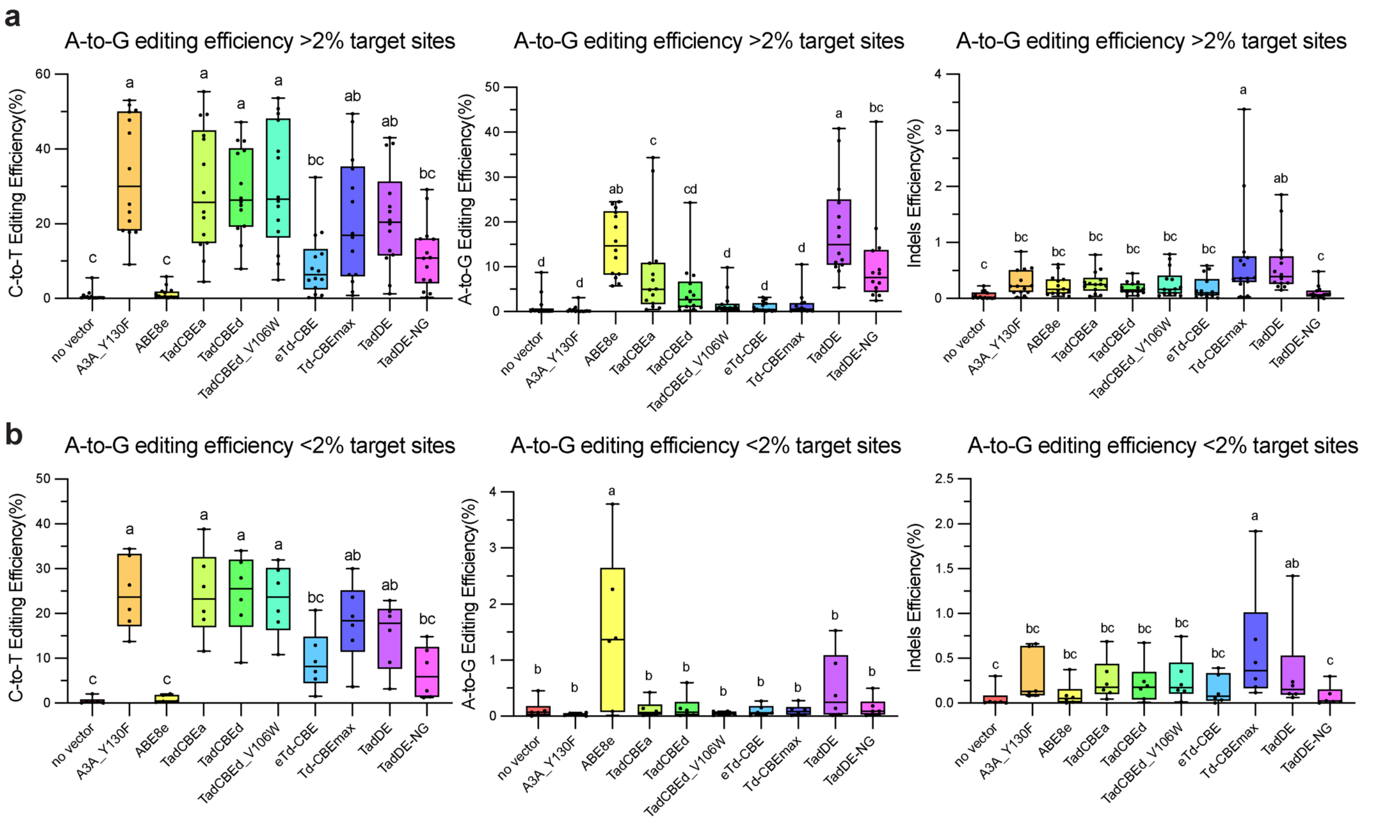

Supplementary Figure 4. Analysis of target sites grouped based on a 2% A-to-G editing efficiency threshold as the breakpoint. (a) For target sites where the A-to-G editing efficiency of TadDE is > 2%, the editing efficiencies of C-to-T, A-to-G, and indels are compared for both TadCBEs and TadDE. **(b)** For target sites where the A-to-G editing efficiency of TadDE is < 2%, the editing efficiencies of C-to-T, A-to-G, and indels are compared for both TadCBEs and TadDE.

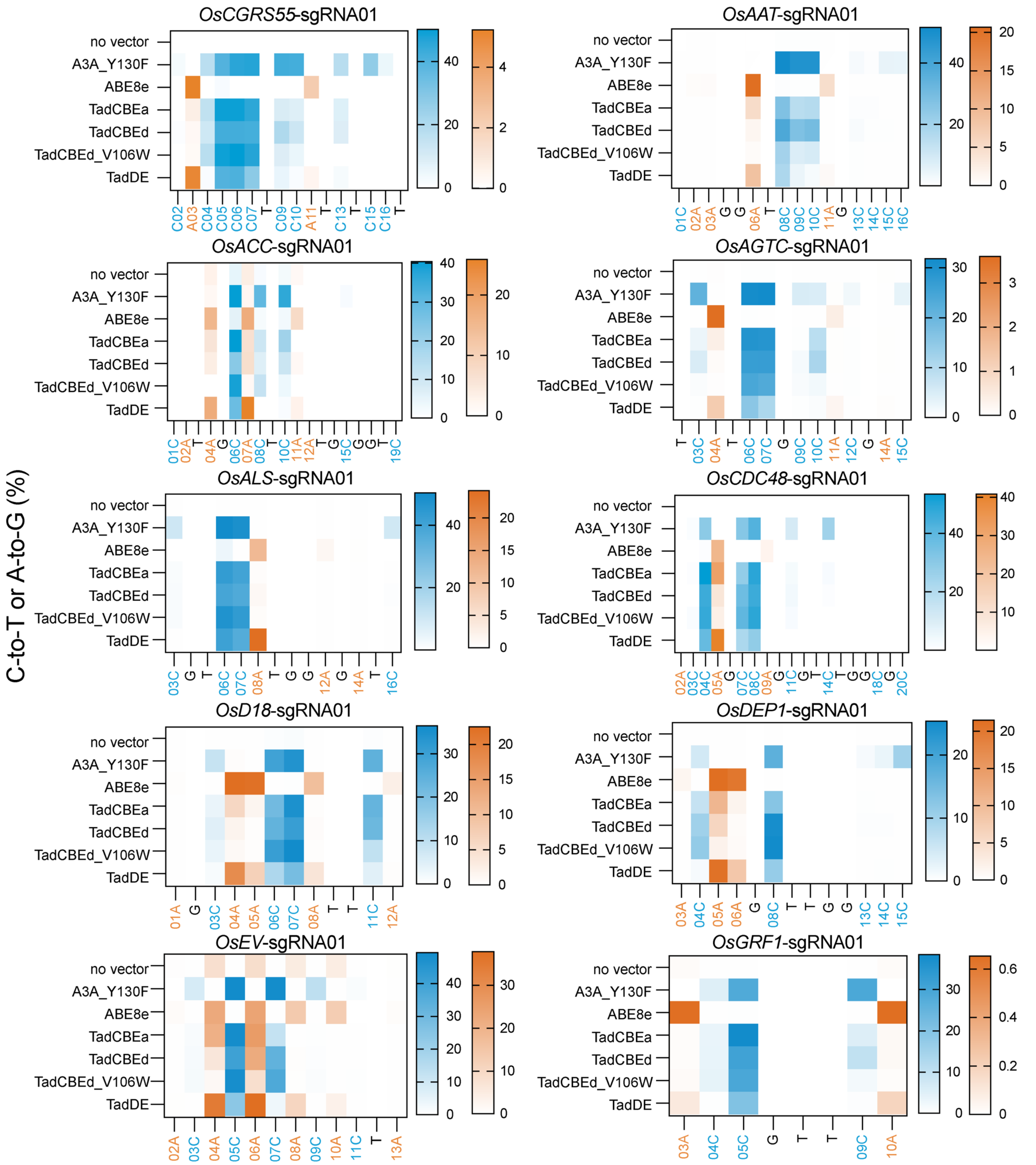

Supplementary Figure 5. Editing profiles, including efficiency, windows, and sequence preferences in multiplex array 01 target sites in rice protoplasts. Heatmaps display the on-target C-to-T and A-to-G editing efficiencies and windows of A3A_Y130F, ABE8e, TadCBEa, TadCBEd, TadCBEd_V106W, and TadDE at 10 gRNA array 01 target sites in rice cells.

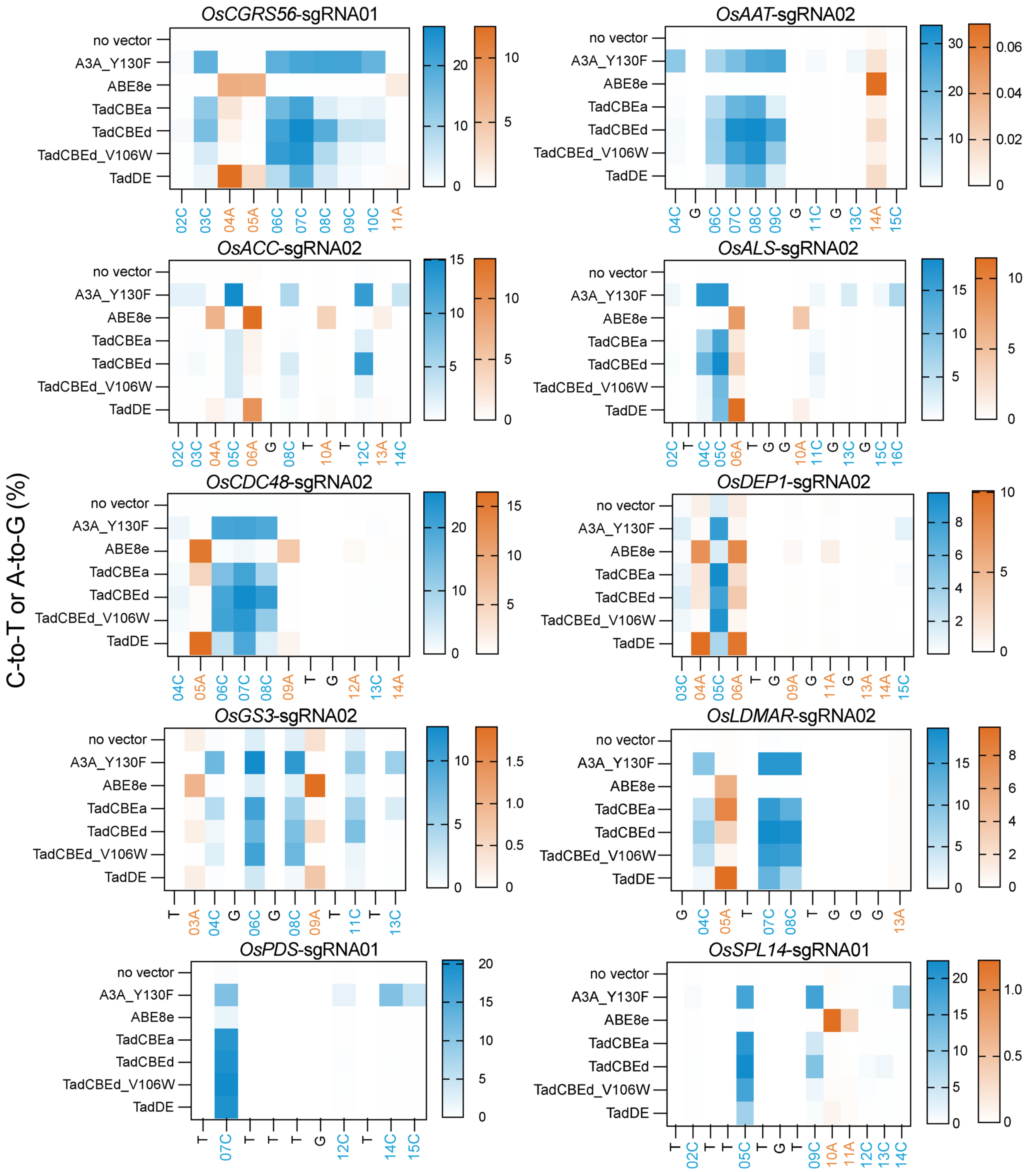

Supplementary Figure 6. Editing profiles, including efficiency, windows, and sequence preferences in multiplex array 02 target sites in rice protoplasts. Heatmaps display the on-target C-to-T and A-to-G editing efficiencies and windows of A3A_Y130F, ABE8e, TadCBEa, TadCBEd, TadCBEd_V106W, and TadDE at 10 gRNA array 02 target sites in rice cells.

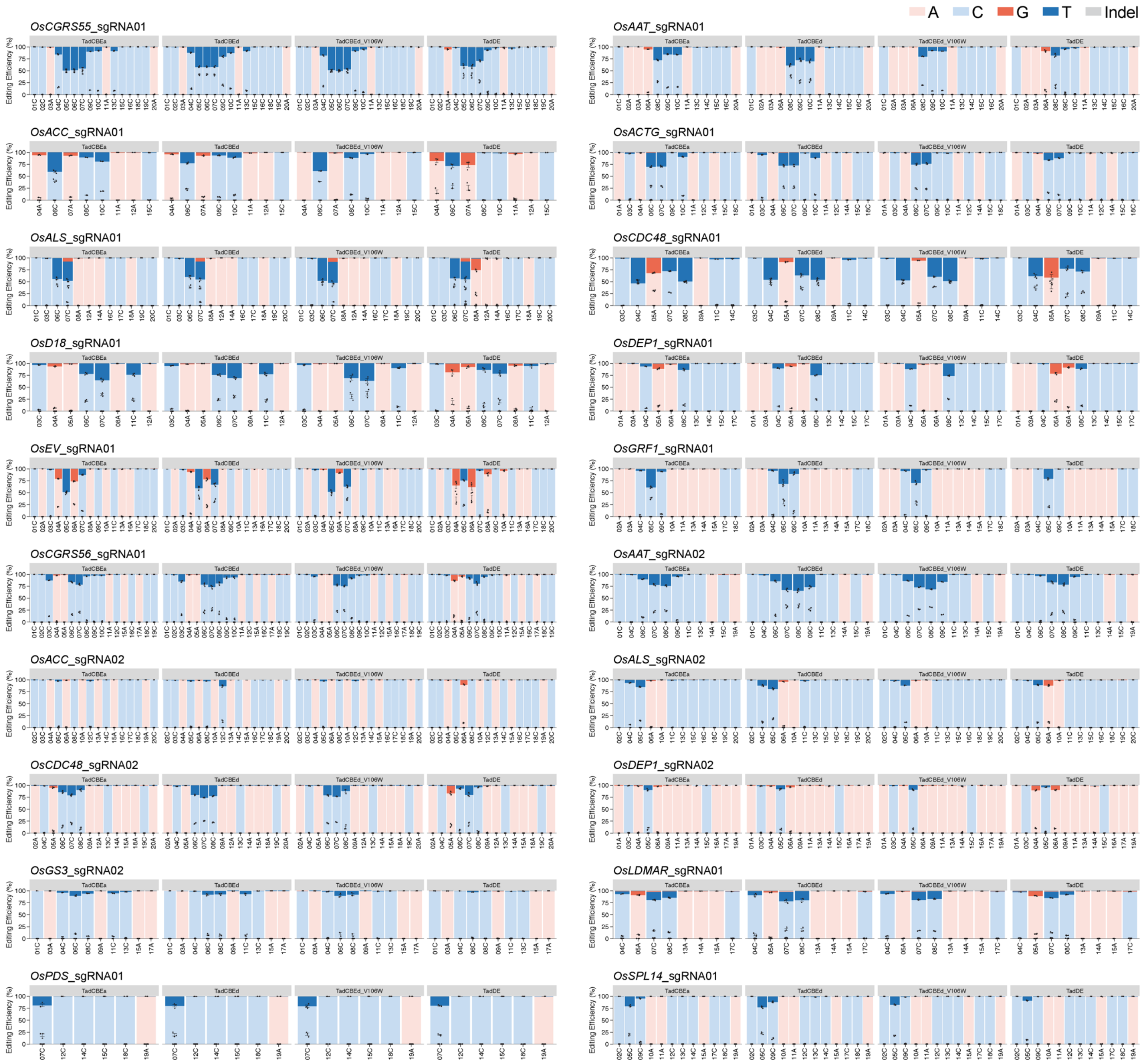

Supplementary Figure 7. Base editing preferences at each A and C nucleotide among 20 sgRNA-targeted sites in rice protoplasts treated with TadCBEa, TadCBEd, TadCBEd_V106W, and TadDE. Bar plots depict on-target DNA base editing frequencies and windows of cytosines with C-to-T edits and adenines with A-to-G edits at 20 target sites in rice cells. Each bar represents the average of three independent replicates (n = 3) at each base.

***OsCGRS55*-sgRNA01**

Protospacer PAM	Ratio	Reads
CCACCCCTCCATCTCCTCCACGG		
CCACCCCTCCATCTCCTCCACGG	38.28%	15296
CCACtttTCCATCTCCTCCACGG	24.96%	9972
CCAttttTCCATCTCCTCCACGG	6.81%	2720
CCACttCTCCATCTCCTCCACGG	3.36%	1344
CCACtttTtCATCTCCTCCACGG	3.05%	1218
CCAttttTCCATtTCCTCCACGG	2.50%	1000
CCACtttTCCATtTCCTCCACGG	2.25%	899
CCACtttTCtATCTCCTCCACGG	2.02%	808
CCAttttTtCATCTCCTCCACGG	1.77%	708
CCACtttTttATCTCCTCCACGG	1.59%	635

***OsAAT*-sgRNA01**

Protospacer PAM	Ratio	Reads
CAAGGATCCCAGCCCCGTGAAGG		
CAAGGATCCCAGCCCCGTGAAGG	67.82%	31628
CAAGGATtttAGCCCCGTGAAGG	9.69%	4517
CAAGGATtCCAGCCCCGTGAAGG	7.69%	3586
CAAGGATttCAGCCCCGTGAAGG	3.04%	1416
CAAGGATtCtAGCCCCGTGAAGG	3.00%	1397
CAAGGgTtttAGCCCCGTGAAGG	1.96%	912
CAAGGgTtCCAGCCCCGTGAAGG	0.98%	455
CAAGGgTtCtAGCCCCGTGAAGG	0.92%	429
CAAGGgTttCAGCCCCGTGAAGG	0.62%	291
CAAGGATCCtAGCCCCGTGAAGG	0.41%	192

***OsACC*-sgRNA01**

Protospacer PAM	Ratio	Reads
CATAGCACTCAATGCGGTCTGGG		
CATAGCACTCAATGCGGTCTGGG	53.46%	12904
CATAGtACTCAATGCGGTCTGGG	16.46%	3973
CATAGtAtTtAATGCGGTCTGGG	7.82%	1887
CATAGtACTtAATGCGGTCTGGG	4.71%	1138
CATAGtgCTCAATGCGGTCTGGG	3.33%	803
CATgGtACTCAATGCGGTCTGGG	3.10%	749
CATAGCACTtAATGCGGTCTGGG	1.55%	375
CATAGtgCTtAATGCGGTCTGGG	1.14%	276
CATgGtACTtAATGCGGTCTGGG	1.12%	271
CATAGCAtTtAATGCGGTCTGGG	0.77%	185

***OsACTG*-sgRNA01**

Protospacer PAM	Ratio	Reads
ATCATCCGCCACGACGGCGGCGG		
ATCATCCGCCACGACGGCGGCGG	62.71%	29355
ATCATttGCCACGACGGCGGCGG	17.86%	8360
ATCATttGCtACGACGGCGGCGG	7.62%	3567
ATtATttGCCACGACGGCGGCGG	1.39%	652
ATtATttGCtACGACGGCGGCGG	1.29%	602
ATCATtCGCCACGACGGCGGCGG	0.95%	445
ATCATttGCCACGACGGCGGCGG	0.27%	128
ATCATCtGCCACGACGGCGGCGG	0.17%	81
ATCgTttGCCACGACGGCGGCGG	0.17%	78
ATCATtCGCCACGACGGCGGCGG	0.13%	63

***OsALS*-sgRNA01**

Protospacer PAM	Ratio	Reads
CGCGTCCATGGAGATCCACCAGG		
CGCGTCCATGGAGATCCACCAGG	46.01%	21972
CGCGTttATGGAGATCCACCAGG	39.59%	18905
CGCGTCgATGGAGATCCACCAGG	6.07%	2899
CGCGTtgATGGAGATCCACCAGG	2.95%	1407
CGCGTtCATGGAGATCCACCAGG	1.12%	536
CGtGTttATGGAGATCCACCAGG	1.08%	518
CGCGTttgTGGAGATCCACCAGG	0.34%	160
CGCGTttATGGAGATCCACCAGG	0.18%	85
CGCGTCtATGGAGATCCACCAGG	0.16%	77
CGtGTtgATGGAGATCCACCAGG	0.09%	42

***OsCDC48*-sgRNA01**

Protospacer PAM	Ratio	Reads
GACCAGCCAGCGTCTGGCGCCGG		
GACCAGCCAGCGTCTGGCGCCGG	41.11%	19734
GACtgGttAGCGTCTGGCGCCGG	17.87%	8579
GACtAGCtAGCGTCTGGCGCCGG	11.36%	5454
GACtgGCtAGCGTCTGGCGCCGG	8.82%	4234
GACtAGttAGCGTCTGGCGCCGG	6.31%	3028
GACtAGCCAGCGTCTGGCGCCGG	2.83%	1357
GACtgGCCAGCGTCTGGCGCCGG	2.17%	1044
GACtgGttAGtGTCTGGCGCCGG	1.32%	635
GACCAGCtAGCGTCTGGCGCCGG	0.85%	408
GACtgGttAGCGTtTGGCGCCGG	0.83%	398

***OsD18*-sgRNA01**

Protospacer PAM	Ratio	Reads
AGCAACCATTCATGGATGGATGG		
AGCAACCATTCATGGATGGATGG	33.86%	8767
AGCAAttATTtATGGATGGATGG	14.59%	3778
AGCAACtATTtATGGATGGATGG	12.21%	3161
AGCAAttATTCATGGATGGATGG	7.98%	2065
AGCAACtATTCATGGATGGATGG	7.78%	2014
AGCgAttATTtATGGATGGATGG	3.92%	1015
AGCgAttATTCATGGATGGATGG	1.75%	454
AGtAAttATTtATGGATGGATGG	1.43%	371
AGCAACCATTtATGGATGGATGG	1.04%	270
AGCgACtATTtATGGATGGATGG	0.99%	256

***OsDEP1*-sgRNA01**

Protospacer PAM	Ratio	Reads
AGACAAGCTTGGCCCTCTTTGGG		
AGACAAGCTTGGCCCTCTTTGGG	75.87%	34790
AGACgAGtTTGGCCCTCTTTGGG	4.43%	2030
AGACAAGtTTGGCCCTCTTTGGG	2.91%	1336
AGACgAGCTTGGCCCTCTTTGGG	2.65%	1216
AGAtgAGtTTGGCCCTCTTTGGG	2.29%	1051
AGAtAAGtTTGGCCCTCTTTGGG	1.54%	706
AGAtgAGCTTGGCCCTCTTTGGG	0.83%	379
AGAtAAGCTTGGCCCTCTTTGGG	0.73%	337
AGACggGtTTGGCCCTCTTTGGG	0.64%	293
AGAtggGtTTGGCCCTCTTTGGG	0.41%	188

***OsEV*-sgRNA01**

Protospacer PAM	Ratio	Reads
CACACACACACTAGTACCTCTGG		
CACACACACACTAGTACCTCTGG	39.83%	3812
CACAtACACACTAGTACCTCTGG	12.00%	1148
CACAtgCACACTAGTACCTCTGG	10.22%	978
CACgtgCACACTAGTACCTCTGG	7.57%	724
CACgtACACACTAGTACCTCTGG	6.34%	607
CACgtgtACACTAGTACCTCTGG	4.17%	399
CACAtgtACACTAGTACCTCTGG	2.92%	279
CACAtAtACACTAGTACCTCTGG	2.04%	195
CACgtAtACACTAGTACCTCTGG	1.00%	96
CACACACACACTAGTACCTCTGG	0.65%	62

***OsGRF1*-sgRNA01**

Protospacer PAM	Ratio	Reads
GAACCGTTCAAGAAAGCCTGTGG		
GAACCGTTCAAGAAAGCCTGTGG	54.43%	20919
GAACtGTTCAAGAAAGCCTGTGG	31.82%	12228
GAACtGTTtAAGAAAGCCTGTGG	4.62%	1776
GAAttGTTCAAGAAAGCCTGTGG	2.81%	1080
GAAttGTTtAAGAAAGCCTGTGG	0.94%	361
GAACtGTTCAAGAAAGCCTGTGG	0.66%	255
GAACCGTTtAAGAAAGCCTGTGG	0.35%	136
GAACCGTTCAAGAAAGCCTGTGG	0.23%	89
GAACtGTTCAAGAAAGCCTGTGG	0.08%	30
GAACtGTTtAAGAAAGCCTGTGG	0.05%	21

***OsCGRS56*-sgRNA01**

Protospacer PAM	Ratio	Reads
CCCAACCCCCACTGACACCTAGG		
CCCAACCCCCACTGACACCTAGG	58.64%	12387
CCtAAttCCCACTGACACCTAGG	5.69%	1203
CCCAAttCCCACTGACACCTAGG	5.43%	1147
CCCAACtCCCACTGACACCTAGG	2.90%	612
CCtAACtCCCACTGACACCTAGG	2.67%	565
CCtAAtttCCACTGACACCTAGG	1.29%	272
CCtAACCCCCACTGACACCTAGG	0.83%	175
CCtAAtCCCCACTGACACCTAGG	0.75%	158
CCCAAtttCCACTGACACCTAGG	0.67%	141
CCtAAttCCtACTGACACCTAGG	0.56%	118

***OsAAT*-sgRNA02**

Protospacer PAM	Ratio	Reads
CTGCGCCCCGCGCACGTTAGCGG		
CTGCGCCCCGCGCACGTTAGCGG	72.19%	33808
CTGCGCttCGCGCACGTTAGCGG	8.74%	4095
CTGCGtttCGCGCACGTTAGCGG	6.18%	2892
CTGCGCCtCGCGCACGTTAGCGG	2.82%	1322
CTGCGCtttGCGCACGTTAGCGG	2.19%	1026
CTGCGtCtCGCGCACGTTAGCGG	1.30%	610
CTGCGCtCCGCGCACGTTAGCGG	1.08%	508
CTGCGttttGCGCACGTTAGCGG	0.93%	435
CTGCGCCttGCGCACGTTAGCGG	0.44%	208
CTGCGttCCGCGCACGTTAGCGG	0.42%	198

***OsACC*-sgRNA02**

Protospacer PAM	Ratio	Reads
TCCACAGCTATCACACCCACTGG		
TCCACAGCTATCACACCCACTGG	91.66%	42139
TCCAtAGCTATCACACCCACTGG	2.16%	992
TCCACAGCTATtACACCCACTGG	1.69%	776
TCCACgGCTATCACACCCACTGG	0.49%	225
TCCAtgGCTATCACACCCACTGG	0.40%	183
TCCAtAGCTATtACACCCACTGG	0.31%	143
TCCACgGCTATtACACCCACTGG	0.12%	57
TCCACAGtTATCACACCCACTGG	0.12%	53
TCCAtgGCTATtACACCCACTGG	0.08%	36
TCCACAGtTATtACACCCACTGG	0.07%	32

***OsALS*-sgRNA02**

Protospacer PAM	Ratio	Reads
TCTCCATGGACGCGCCGCCCGGG		
TCTCCATGGACGCGCCGCCCGGG	80.00%	38111
TCTCtATGGACGCGCCGCCCGGG	6.61%	3150
TCTttATGGACGCGCCGCCCGGG	4.92%	2346
TCTCtgTGGACGCGCCGCCCGGG	0.93%	444
TCTttgTGGACGCGCCGCCCGGG	0.89%	425
TCTCtATGGAtGCGCCGCCCGGG	0.30%	145
TCTttATGGAtGCGCCGCCCGGG	0.21%	99
TCTCtgTGGAtGCGCCGCCCGGG	0.10%	47
TCTCCATGGAtGCGCCGCCCGGG	0.07%	34
TCTCtATGGACGCGCCGCCCGGG	0.03%	15

***OsCDC48*-sgRNA02**

Protospacer PAM	Ratio	Reads
TAGCACCCATGACAATGACATGG		
TAGCACCCATGACAATGACATGG	71.67%	33862
TAGCAttCATGACAATGACATGG	7.57%	3579
TAGCACtCATGACAATGACATGG	4.15%	1960
TAGCACttATGACAATGACATGG	3.11%	1469
TAGCAtttATGACAATGACATGG	2.81%	1328
TAGCgttCATGACAATGACATGG	1.90%	897
TAGCgtttATGACAATGACATGG	1.62%	765
TAGCgCtCATGACAATGACATGG	1.18%	558
TAGCgCttATGACAATGACATGG	0.61%	288
TAGCAtCCATGACAATGACATGG	0.47%	223

***OsDEP1*-sgRNA02**

Protospacer PAM	Ratio	Reads
AGCACATGAGAGAACAATATTGG		
AGCACATGAGAGAACAATATTGG	90.91%	18385
AGCAtATGAGAGAACAATATTGG	3.96%	801
AGCAtgTGAGAGAACAATATTGG	1.29%	261
AGCgtATGAGAGAACAATATTGG	0.78%	157
AGCACATGAGAGAACAATATTGG	0.36%	72
AGtAtATGAGAGAACAATATTGG	0.32%	65
AGCgtgTGAGAGAACAATATTGG	0.30%	61
AGCACATGAGAGAACAATATTGG	0.10%	21
AGCACATGAGAGAACAATATTGG	0.09%	18
AGCACgTGAGAGAACAATATTGG	0.06%	12

***OsGS3*-sgRNA02**

Protospacer PAM	Ratio	Reads
CTACGCGCATCTCTATATTTCGG		
CTACGCGCATCTCTATATTTCGG	84.53%	10751
CTACGtGCATCTCTATATTTCGG	3.34%	425
CTACGtGCATtTCTATATTTCGG	1.79%	228
CTACGtGtATCTCTATATTTCGG	1.35%	172
CTACGCGCATtTCTATATTTCGG	0.97%	123
CTAtGtGtATCTCTATATTTCGG	0.84%	107
CTAtGtGCATCTCTATATTTCGG	0.75%	95
CTAtGtGtATCTtTATATTTCGG	0.71%	90
CTACGtGtATtTCTATATTTCGG	0.64%	82
CTAtGtGtATtTtTATATTTCGG	0.64%	81

***OsLDMAR*-sgRNA01**

Protospacer PAM	Ratio	Reads
GTGCATCCTGGGAAAGCTTGTGG		
GTGCATCCTGGGAAAGCTTGTGG	68.44%	31467
GTGCATttTGGGAAAGCTTGTGG	6.14%	2821
GTGCgTttTGGGAAAGCTTGTGG	3.43%	1578
GTGtgTttTGGGAAAGCTTGTGG	2.85%	1308
GTGtgTtCTGGGAAAGCTTGTGG	1.03%	473
GTGCATtCTGGGAAAGCTTGTGG	0.98%	449
GTGCgTtCTGGGAAAGCTTGTGG	0.85%	390
GTGtATttTGGGAAAGCTTGTGG	0.75%	346
GTGCATCCTGGGAAAGCTTGTGG	0.73%	337
GTGtATtCTGGGAAAGCTTGTGG	0.60%	274

***OsPDS*-sgRNA01**

Protospacer PAM	Ratio	Reads
GTTGGTCTTTGCTCCTGCAGAGG		
GTTGGTCTTTGCTCCTGCAGAGG	84.03%	17743
GTTGGTtTTTGCTCCTGCAGAGG	13.17%	2781
GTTGGTCTTTGCTCCTGCAGAGG	0.09%	20
GTTGGTCTTTGCTCCTGCAGAGG	0.09%	19
GTTGGTCTTTGCTCCTGCAGAGG	0.08%	16
GTTGGTCTTTGCTCCTGCAGAGG	0.07%	14
GTTGGTCTTTGCTCCTGCAGAGG	0.07%	14
GTTGGTCTTTGCTCCTGCAGAGG	0.05%	10
GTTGGTCTTTGCTCCTGCAGAGG	0.04%	9
GTTGGTCTTTGCTCCTGCAGAGG	0.04%	9

***OsSPL14*-sgRNA01**

Protospacer PAM	Ratio	Reads
TCTTCTGTCAACCCAGCCATGGG		
TCTTCTGTCAACCCAGCCATGGG	79.64%	6665
TCTTtTGTCAACCCAGCCATGGG	14.85%	1243
TCTTtTGTtAACCCAGCCATGGG	2.94%	246
TCTTCTGTCAACCCAGCCATGGG	0.23%	19
TCTTCTGTtAACCCAGCCATGGG	0.22%	18
TCTTCTGTCAACCCAGCCATGGG	0.07%	6
TCTTtTGTtAACtCAGCCATGGG	0.06%	5
TCTTCTGTCAACCCAGCCATGGG	0.06%	5
TCTTCTGTCAACCtAGCCATGGG	0.05%	4
TCTTtTGTCAACtCAGCCATGGG	0.04%	3

Supplementary Figure 8. Genotyping data of the most frequent reads at the 20 target sites in rice protoplasts treated with TadCBEa. Sequences in lowercase red indicate base editing outcomes. The values on the right represent the ratio and reads of mutation alleles.

***OsCGRS55*-sgRNA01**

Protospacer PAM

CCACCCCTCCATCTCCTCCACGG	Ratio	Reads
CCACCCCTCCATCTCCTCCACGG	44.36%	17951
CCACtttTCCATCTCCTCCACGG	17.62%	7131
CCACtttTtCATCTCCTCCACGG	6.44%	2608
CCAttttTttATCTCCTCCACGG	3.92%	1586
CCACtttTttATCTCCTCCACGG	3.87%	1568
CCAttttTttATtTCCTCCACGG	3.70%	1497
CCAttttTtCATCTCCTCCACGG	2.75%	1113
CCAttttTCCATCTCCTCCACGG	2.36%	955
CCACtttTCtATCTCCTCCACGG	1.57%	634
CCACtttTCCATtTCCTCCACGG	1.47%	594

***OsAAT*-sgRNA01**

Protospacer PAM

CAAGGATCCCAGCCCCGTGAAGG	Ratio	Reads
CAAGGATCCCAGCCCCGTGAAGG	54.86%	25845
CAAGGATtttAGCCCCGTGAAGG	26.54%	12504
CAAGGATtCCAGCCCCGTGAAGG	6.25%	2945
CAAGGATtCtAGCCCCGTGAAGG	4.49%	2113
CAAGGATttCAGCCCCGTGAAGG	2.01%	946
CAAGGATtttAGtCCCGTGAAGG	1.11%	525
CAAGGgTtttAGCCCCGTGAAGG	0.96%	453
CAAGGgTCCCAGCCCCGTGAAGG	0.28%	130
CAAGGgTtCtAGCCCCGTGAAGG	0.20%	95
CAAGGATtCtAGtCCCGTGAAGG	0.17%	79

***OsACC*-sgRNA01**

Protospacer PAM

CATAGCACTCAATGCGGTCTGGG	Ratio	Reads
CATAGCACTCAATGCGGTCTGGG	67.54%	20641
CATAGtACTCAATGCGGTCTGGG	9.92%	3031
CATAGtACTtAATGCGGTCTGGG	4.54%	1389
CATAGtAtTtAATGCGGTCTGGG	3.47%	1062
CATgGCgCTCAATGCGGTCTGGG	1.82%	557
CATAGtgCTCAATGCGGTCTGGG	1.45%	443
CATAGtgtTtAATGCGGTCTGGG	1.42%	435
CATAGtAtTCAATGCGGTCTGGG	1.35%	413
CATgGCgCTCgATGCGGTCTGGG	1.14%	348
CATAGtgCTtAATGCGGTCTGGG	1.04%	317

***OsACTG*-sgRNA01**

Protospacer PAM

ATCATCCGCCACGACGGCGGCGG	Ratio	Reads
ATCATCCGCCACGACGGCGGCGG	62.93%	29237
ATCATttGCCACGACGGCGGCGG	13.36%	6205
ATCATttGCtACGACGGCGGCGG	8.19%	3807
ATtATttGCtACGACGGCGGCGG	2.87%	1332
ATtATttGCCACGACGGCGGCGG	2.19%	1017
ATCATCCGCCACGACGGCGGCGG	1.38%	641
ATCATttGttACGACGGCGGCGG	0.66%	307
ATCCTCCGCCACGACGGCGGCGG	0.65%	303
ATCATtCGCCACGACGGCGGCGG	0.46%	213
ATtATttGttACGACGGCGGCGG	0.37%	174

***OsALS*-sgRNA01**

Protospacer PAM

CGCGTCCATGGAGATCCACCAGG	Ratio	Reads
CGCGTCCATGGAGATCCACCAGG	47.09%	22612
CGCGTttATGGAGATCCACCAGG	37.14%	17836
CGCGTCgATGGAGATCCACCAGG	6.77%	3250
CGCGTtgATGGAGATCCACCAGG	2.98%	1431
CGtGTttATGGAGATCCACCAGG	1.39%	669
CGCGTttgTGGAGATCCACCAGG	1.07%	512
CGCGTttATGGAGATtCACCAGG	0.39%	187
CGtGTtgATGGAGATCCACCAGG	0.14%	67
CGCGTCtATGGAGATCCACCAGG	0.10%	50
CGCGTgtATGGAGATCCACCAGG	0.07%	33

***OsCDC48*-sgRNA01**

Protospacer PAM

GACCAGCCAGCGTCTGGCGCCGG	Ratio	Reads
GACCAGCCAGCGTCTGGCGCCGG	46.37%	22138
GACtAGttAGCGTCTGGCGCCGG	26.79%	12790
GACtAGCtAGCGTCTGGCGCCGG	8.02%	3830
GACtgGttAGCGTCTGGCGCCGG	7.41%	3537
GACtAGttAGtGTCTGGCGCCGG	2.79%	1330
GACtgGttAGtGTCTGGCGCCGG	1.36%	647
GACtAGCCAGCGTCTGGCGCCGG	1.08%	514
GACCAGttAGCGTCTGGCGCCGG	0.65%	309
GACtgGCtAGCGTCTGGCGCCGG	0.51%	243
GACCAGCtAGCGTCTGGCGCCGG	0.49%	234

***OsD18*-sgRNA01**

Protospacer PAM

AGCAACCATTCATGGATGGATGG	Ratio	Reads
AGCAACCATTCATGGATGGATGG	47.98%	7788
AGCAAttATTtATGGATGGATGG	20.82%	3379
AGCAAttATTCATGGATGGATGG	6.67%	1082
AGCAACtATTtATGGATGGATGG	6.20%	1006
AGtAAttATTtATGGATGGATGG	5.19%	842
AGCAACtATTCATGGATGGATGG	2.61%	423
AGCgAttATTtATGGATGGATGG	1.09%	177
AGtAAttATTCATGGATGGATGG	0.81%	132
AGtgAttATTtATGGATGGATGG	0.75%	121
AGCAACCATTCATGGATGGATGG	0.41%	66

***OsDEP1*-sgRNA01**

Protospacer PAM

AGACAAGCTTGGCCCTCTTTGGG	Ratio	Reads
AGACAAGCTTGGCCCTCTTTGGG	68.14%	31847
AGACAAGtTTGGCCCTCTTTGGG	11.57%	5406
AGAtAAGtTTGGCCCTCTTTGGG	7.40%	3461
AGACgAGtTTGGCCCTCTTTGGG	3.19%	1493
AGAtgAGtTTGGCCCTCTTTGGG	2.86%	1339
AGACAAGtTTGGtCCTCTTTGGG	0.24%	112
AGACggGtTTGGCCCTCTTTGGG	0.15%	69
AGAtAAGtTTGGtCCTCTTTGGG	0.15%	69
AGACgAGCTTGGCCCTCTTTGGG	0.11%	51
AGAtAAGCTTGGCCCTCTTTGGG	0.09%	43

***OsEV*-sgRNA01**

Protospacer PAM

CACACACACACTAGTACCTCTGG	Ratio	Reads
CACACACACACTAGTACCTCTGG	45.20%	3735
CACAtgtACACTAGTACCTCTGG	14.46%	1195
CACAtAtACACTAGTACCTCTGG	11.92%	985
CACAtACACACTAGTACCTCTGG	5.57%	460
CACgtgtACACTAGTACCTCTGG	3.92%	324
CACAtgCACACTAGTACCTCTGG	3.10%	256
CACgtAtACACTAGTACCTCTGG	1.59%	131
CACACAtACACTAGTACCTCTGG	1.04%	86
CAtAtgtACACTAGTACCTCTGG	0.73%	60
CACACgtACACTAGTACCTCTGG	0.69%	57

***OsGRF1*-sgRNA01**

Protospacer PAM

GAACCGTTCAAGAAAGCCTGTGG	Ratio	Reads
GAACCGTTCAAGAAAGCCTGTGG	56.69%	26342
GAACtGTTCAAGAAAGCCTGTGG	22.81%	10599
GAACtGTTtAAGAAAGCCTGTGG	10.08%	4686
GAAttGTTCAAGAAAGCCTGTGG	2.62%	1218
GAAttGTTtAAGAAAGCCTGTGG	2.44%	1135
GAACCGTTtAAGAAAGCCTGTGG	0.58%	269
GAACtGTTCAAGAAAGCCTGTGG	0.08%	38
GAACCGTTCAAGAAAGCCTGTGG	0.06%	29
GAACtGTTCAAGAAAGCCTGTGG	0.06%	29
GAACtGTTCAAGAAAGCCTGTGG	0.06%	26

***OsCGRS56*-sgRNA01**

Protospacer PAM

CCCAACCCCCACTGACACCTAGG	Ratio	Reads
CCCAACCCCCACTGACACCTAGG	51.18%	10670
CCtAAtttCCACTGACACCTAGG	4.78%	996
CCCAAtttCCACTGACACCTAGG	4.37%	911
CCCAAttCCCACTGACACCTAGG	4.22%	879
CCtAAttCCCACTGACACCTAGG	2.72%	568
CCtAAttttCACTGACACCTAGG	2.44%	508
CCtAAtttttACTGACACCTAGG	2.02%	422
CCCAACtCCCACTGACACCTAGG	1.58%	329
CCCAACttCCACTGACACCTAGG	1.42%	297
CCtAAtttCtACTGACACCTAGG	1.13%	236

***OsAAT*-sgRNA02**

Protospacer PAM

CTGCGCCCCGCGCACGTTAGCGG	Ratio	Reads
CTGCGCCCCGCGCACGTTAGCGG	62.58%	11344
CTGCGCtttGCGCACGTTAGCGG	16.71%	3029
CTGCGttttGCGCACGTTAGCGG	8.29%	1503
CTGCGtttCGCGCACGTTAGCGG	4.12%	746
CTGCGCttCGCGCACGTTAGCGG	2.69%	488
CTGtGttttGCGCACGTTAGCGG	0.73%	132
CTGCGCCttGCGCACGTTAGCGG	0.51%	92
CTGCGCCtCGCGCACGTTAGCGG	0.39%	70
CTGtGCtttGCGCACGTTAGCGG	0.24%	44
CTGCGCCCCGCGCACGTTAGCGG	0.20%	37

***OsACC*-sgRNA02**

Protospacer PAM

TCCACAGCTATCACACCCACTGG	Ratio	Reads
TCCACAGCTATCACACCCACTGG	84.44%	39240
TCCACAGCTATtACACCCACTGG	7.80%	3624
TCCACAGtTATtACACCCACTGG	1.31%	611
TCCAtAGCTATtACACCCACTGG	1.10%	509
TCCAtAGCTATCACACCCACTGG	0.62%	290
TCCAtAGtTATtACACCCACTGG	0.32%	147
TCCACgGCTATtACACCCACTGG	0.27%	125
TCCACAGtTATCACACCCACTGG	0.27%	124
TCCACgGCTATCACACCCACTGG	0.26%	119
TCCACAGCTATCACACCCACTGG	0.23%	109

***OsALS*-sgRNA02**

Protospacer PAM

TCTCCATGGACGCGCCGCCCGGG	Ratio	Reads
TCTCCATGGACGCGCCGCCCGGG	57.63%	26964
TCTttATGGACGCGCCGCCCGGG	6.18%	2893
TCTCtATGGACGCGCCGCCCGGG	3.47%	1622
TCTttgTGGACGCGCCGCCCGGG	2.01%	941
TCTCtgTGGACGCGCCGCCCGGG	0.57%	268
TCTttATGGAtGCGCCGCCCGGG	0.57%	266
TCTCtATGGAtGCGCCGCCCGGG	0.53%	249
TCTttATGGACGCGCCGCCCGGG	0.25%	119
TCTttATGGACGCGCCGCCCGGG	0.17%	79
TCTttgTGGAtGCGCCGCCCGGG	0.15%	68

***OsCDC48*-sgRNA02**

Protospacer PAM

TAGCACCCATGACAATGACATGG	Ratio	Reads
TAGCACCCATGACAATGACATGG	69.10%	4877
TAGCAtttATGACAATGACATGG	14.20%	1023
TAGCACttATGACAATGACATGG	5.23%	377
TAGCAttCATGACAATGACATGG	2.61%	188
TAGCACtCATGACAATGACATGG	1.68%	121
TAGtAtttATGACAATGACATGG	1.18%	85
TAGtACttATGACAATGACATGG	0.36%	26
TAGCgtttATGACAATGACATGG	0.29%	21
TAGtAttCATGACAATGACATGG	0.19%	14
TAGtgtttATGACAATGACATGG	0.15%	11

***OsDEP1*-sgRNA02**

Protospacer PAM

AGCACATGAGAGAACAATATTGG	Ratio	Reads
AGCACATGAGAGAACAATATTGG	89.04%	22100
AGCAtATGAGAGAACAATATTGG	3.84%	953
AGCAtgTGAGAGAACAATATTGG	2.08%	517
AGCgtATGAGAGAACAATATTGG	0.57%	141
AGtAtATGAGAGAACAATATTGG	0.56%	140
AGCgCgTGAGAGAACAATATTGG	0.51%	127
AGtAtgTGAGAGAACAATATTGG	0.26%	64
AGCgtgTGAGAGAACAATATTGG	0.17%	43
AGCACgTGAGAGAACAATATTGG	0.10%	25
AGtgtATGAGAGAACAATATTGG	0.07%	18

***OsGS3*-sgRNA02**

Protospacer PAM

CTACGCGCATCTCTATATTTCGG	Ratio	Reads
CTACGCGCATCTCTATATTTCGG	88.55%	4917
CTACGtGtATtTCTATATTTCGG	3.87%	215
CTACGtGtATCTCTATATTTCGG	1.10%	61
CTACGtGCATtTCTATATTTCGG	0.86%	48
CTAtGtGtATtTCTATATTTCGG	0.76%	42
CTACGCGCgTCTCTATATTTCGG	0.52%	29
CTACGCGCATtTCTATATTTCGG	0.47%	26
CTACGCGtATCTCTATATTTCGG	0.23%	13
CTACGtGCATCTCTATATTTCGG	0.23%	13
CTgCGCGCgTCTCTATATTTCGG	0.20%	11

***OsLDMAR*-sgRNA01**

Protospacer PAM

GTGCATCCTGGGAAAGCTTGTGG	Ratio	Reads
GTGCATCCTGGGAAAGCTTGTGG	69.21%	7678
GTGCATttTGGGAAAGCTTGTGG	7.01%	778
GTGtATttTGGGAAAGCTTGTGG	4.99%	554
GTGtgTttTGGGAAAGCTTGTGG	1.27%	141
GTGCgTttTGGGAAAGCTTGTGG	0.50%	56
GTGCATtCTGGGAAAGCTTGTGG	0.27%	30
GTGtATtCTGGGAAAGCTTGTGG	0.23%	25
GTGCATCCTGGGAAAGCTTGTGG	0.14%	15
GTGCATCCTGGGAAAGCTTGTGG	0.13%	14
GTGCATCCTGGGAAAGCTTGTGG	0.13%	14

***OsPDS*-sgRNA01**

Protospacer PAM

GTTGGTCTTTGCTCCTGCAGAGG	Ratio	Reads
GTTGGTCTTTGCTCCTGCAGAGG	80.51%	12142
GTTGGTtTTTGCTCCTGCAGAGG	16.34%	2465
GTTGGTCTTTGCTCCTGCAGAGG	0.09%	13
GTTGGTtTTTGtTCCTGCAGAGG	0.08%	12
GTTGGTCTTTGCTCCTGCAGAGG	0.08%	12
GTTGGTCTTTGCTCCTGCAGAGG	0.07%	11
GTTGGTCTTTGCTCCTGCAGAGG	0.07%	10
GTTGGTCTTTGCTCCTGCAGAGG	0.07%	10
GTTGGTCTTTGCTCCTGCAGAGG	0.05%	8
GTTGGTCTTTGCTtCTGCAGAGG	0.05%	7

***OsSPL14*-sgRNA01**

Protospacer PAM

TCTTCTGTCAACCCAGCCATGGG	Ratio	Reads
TCTTCTGTCAACCCAGCCATGGG	74.50%	12072
TCTTtTGTtAACCCAGCCATGGG	11.15%	1807
TCTTtTGTCAACCCAGCCATGGG	9.33%	1512
TCTTtTGTtAACtCAGCCATGGG	0.76%	123
TCTTtTGTCAACtCAGCCATGGG	0.62%	100
TCTTtTGTCAAtCCAGCCATGGG	0.39%	63
TCTTCTGTtAACCCAGCCATGGG	0.36%	58
TCTTtTGTtAAtCCAGCCATGGG	0.20%	33
TCTTCTGTCAACtCAGCCATGGG	0.09%	14
TCTTCTGTCAACCCAGCCATGGG	0.08%	13

Supplementary Figure 9. Genotyping data of the most frequent reads at the 20 target sites in rice protoplasts treated with TadCBEd. Sequences in lowercase red indicate base editing outcomes. The values on the right represent the ratio and reads of mutation alleles.

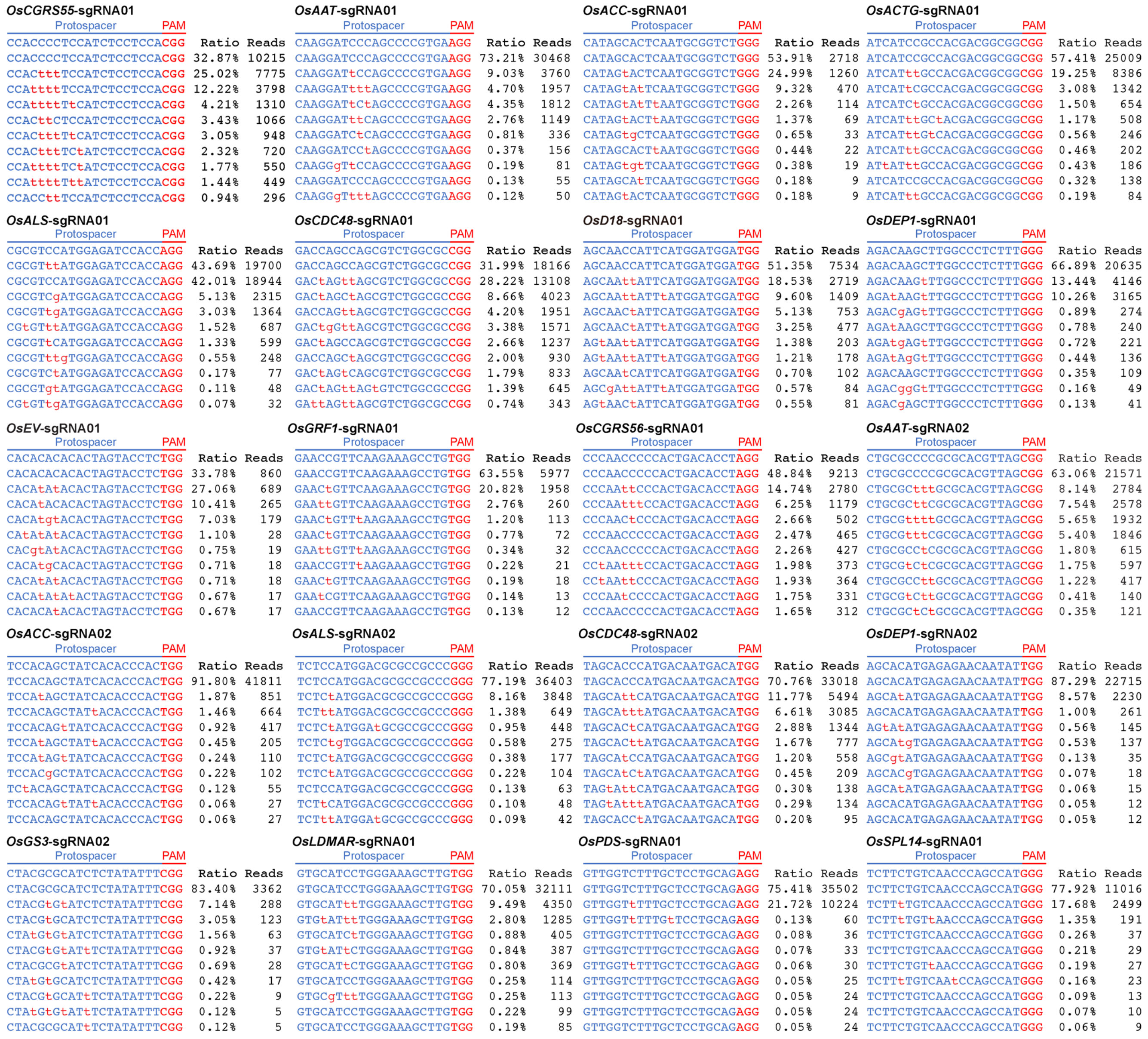

Supplementary Figure 10. Genotyping data of the most frequent reads at the 20 target sites in rice protoplasts treated with TadCBEd_V106W. Sequences in lowercase red indicate base editing outcomes. The values on the right represent the ratio and reads of mutation alleles.

***OsCGRS55*-sgRNA01**

Protospacer PAM

CCACCCCTCCATCTCCTCCACGG	Ratio	Reads
CCACCCCTCCATCTCCTCCACGG	52.26%	13770
CCACtttTCCATCTCCTCCACGG	19.62%	5170
CCACttCTCCATCTCCTCCACGG	10.44%	2750
CCACttttTtCATCTCCTCCACGG	2.73%	718
CCgCtttTCCATCTCCTCCACGG	1.90%	500
CCgCtttTtCATCTCCTCCACGG	0.90%	238
CCACtttTCCATtTCCTCCACGG	0.85%	224
CCgCttCTCCATCTCCTCCACGG	0.61%	160
CCAttttTCCATCTCCTCCACGG	0.60%	159
CCACtttTCtATCTCCTCCACGG	0.54%	141

***OsAAT*-sgRNA01**

Protospacer PAM

CAAGGATCCCAGCCCCGTGAAGG	Ratio	Reads
CAAGGATCCCAGCCCCGTGAAGG	75.87%	30892
CAAGGATtCCAGCCCCGTGAAGG	8.97%	3653
CAAGGgTtCCAGCCCCGTGAAGG	4.97%	2023
CAAGGgTttCAGCCCCGTGAAGG	2.58%	1049
CAAGGATttCAGCCCCGTGAAGG	0.88%	359
CAAGGgTCCCAGCCCCGTGAAGG	0.76%	309
CAAGGgTtCtAGCCCCGTGAAGG	0.62%	253
CAAGGgTtttAGCCCCGTGAAGG	0.45%	184
CAAGGATtCtAGCCCCGTGAAGG	0.42%	170
CAAGGATtttAGCCCCGTGAAGG	0.23%	94

***OsACTG*-sgRNA01**

Protospacer PAM

ATCATCCGCCACGACGGCGGCGG	Ratio	Reads
ATCATCCGCCACGACGGCGGCGG	75.89%	34819
ATCATttGCCACGACGGCGGCGG	8.61%	3951
ATCATtCGCCACGACGGCGGCGG	4.40%	2019
ATCATCCGCCACGACGGCGGCGG	1.67%	764
ATCgTttGCCACGACGGCGGCGG	1.02%	467
ATCATttGCtACGACGGCGGCGG	0.41%	186
ATCgTtCGCCACGACGGCGGCGG	0.32%	146
ATCATttGCCgCGACGGCGGCGG	0.18%	84
ATCATCtGCCACGACGGCGGCGG	0.17%	76
ATCATttGCCACGACGGCGGCGG	0.16%	75

***OsDEP1*-sgRNA01**

Protospacer PAM

AGACAAGCTTGGCCCTCTTTGGG	Ratio	Reads
AGACAAGCTTGGCCCTCTTTGGG	68.49%	31532
AGACgAGCTTGGCCCTCTTTGGG	10.10%	4648
AGACggGtTTGGCCCTCTTTGGG	7.22%	3323
AGACgAGtTTGGCCCTCTTTGGG	5.77%	2657
AGACggGCTTGGCCCTCTTTGGG	2.47%	1139
AGACAAGtTTGGCCCTCTTTGGG	0.43%	200
AGAtggGtTTGGCCCTCTTTGGG	0.23%	105
AGAtgAGCTTGGCCCTCTTTGGG	0.17%	76
AGAtgAGtTTGGCCCTCTTTGGG	0.15%	70
AGAtggGCTTGGCCCTCTTTGGG	0.14%	66

***OsEV*-sgRNA01**

Protospacer PAM

CACACACACACTAGTACCTCTGG	Ratio	Reads
CACACACACACTAGTACCTCTGG	50.76%	7414
CACgCgCACACTAGTACCTCTGG	12.73%	1859
CACgtgCACACTAGTACCTCTGG	6.09%	890
CACAtACACACTAGTACCTCTGG	5.95%	869
CACgtgCgCACTAGTACCTCTGG	3.69%	539
CACgCgCgCACTAGTACCTCTGG	2.48%	362
CACAtgCACACTAGTACCTCTGG	1.16%	169
CACACgCACACTAGTACCTCTGG	1.09%	159
CACgtgCACgCTAGTACCTCTGG	0.94%	137
CACgtgtgCACTAGTACCTCTGG	0.81%	119

***OsGRF1*-sgRNA01**

Protospacer PAM

GAACCGTTCAAGAAAGCCTGTGG	Ratio	Reads
GAACCGTTCAAGAAAGCCTGTGG	74.01%	33336
GAACtGTTCAAGAAAGCCTGTGG	19.72%	8883
GAACtGTTCAAGAAAGCCTGTGG	0.34%	155
GAACtGTTtAAGAAAGCCTGTGG	0.32%	143
GAAttGTTCAAGAAAGCCTGTGG	0.20%	91
GAACtGTTCgAGAAAGCCTGTGG	0.15%	67
GAgCtGTTCAAGAAAGCCTGTGG	0.12%	56
GAACtGTTCAAGAAAGCCTGTGG	0.09%	39
GAAtCGTTCAAGAAAGCCTGTGG	0.08%	38
GAACCGTTCAAGAAAGCCTGTGG	0.07%	33

***OsAAT*-sgRNA02**

Protospacer PAM

CTGCGCCCCGCGCACGTTAGCGG	Ratio	Reads
CTGCGCCCCGCGCACGTTAGCGG	70.52%	16058
CTGCGCttCGCGCACGTTAGCGG	12.50%	2846
CTGCGCtttGCGCACGTTAGCGG	4.21%	958
CTGCGCCtCGCGCACGTTAGCGG	3.92%	893
CTGCGtttCGCGCACGTTAGCGG	1.90%	432
CTGCGtCtCGCGCACGTTAGCGG	1.11%	252
CTGCGCCttGCGCACGTTAGCGG	0.85%	194
CTGCGCtCCGCGCACGTTAGCGG	0.73%	166
CTGCGCtCtGCGCACGTTAGCGG	0.46%	104
CTGCGCCCCGCGCACGTTAGCGG	0.27%	62

***OsACC*-sgRNA02**

Protospacer PAM

TCCACAGCTATCACACCCACTGG	Ratio	Reads
TCCACAGCTATCACACCCACTGG	85.73%	39053
TCCACgGCTATCACACCCACTGG	8.63%	3932
TCCgCgGCTATCACACCCACTGG	0.65%	295
TCCACgGtTATCACACCCACTGG	0.47%	215
TCCACAGCTATCACACCCACTGG	0.38%	173
TCCAtgGCTATCACACCCACTGG	0.38%	171
TCCACgGCTgTCACACCCACTGG	0.34%	153
TCCACgGCTATtACACCCACTGG	0.32%	148
TCCgCAGCTATCACACCCACTGG	0.24%	111

***OsDEP1*-sgRNA02**

Protospacer PAM

AGCACATGAGAGAACAATATTGG	Ratio	Reads
AGCACATGAGAGAACAATATTGG	84.97%	21577
AGCgCgTGAGAGAACAATATTGG	5.94%	1508
AGCgtgTGAGAGAACAATATTGG	3.13%	794
AGCACATGAGAGAACAATATTGG	1.41%	357
AGCgCATGAGAGAACAATATTGG	1.00%	255
AGCACgTGAGAGAACAATATTGG	0.34%	87
AGCAtgTGAGAGAACAATATTGG	0.29%	74
AGCAtATGAGAGAACAATATTGG	0.26%	65
AGCgtgTGAGAGAACAATATTGG	0.07%	19
AGCgtATGAGAGAACAATATTGG	0.06%	15

***OsGS3*-sgRNA02**

Protospacer PAM

CTACGCGCATCTCTATATTTCGG	Ratio	Reads
CTACGCGCATCTCTATATTTCGG	93.35%	18453
CTACGtGCATCTCTATATTTCGG	1.68%	333
CTACGCGCATtTCTATATTTCGG	0.49%	96
CTACGtGtATCTCTATATTTCGG	0.45%	88
CTACGCGCgTCTCTATATTTCGG	0.45%	88
CTACGtGCATtTCTATATTTCGG	0.24%	48
CTACGtGCgTCTCTATATTTCGG	0.18%	36
CTACGtGtATtTCTATATTTCGG	0.16%	32
CTgCGCGCATCTCTATATTTCGG	0.15%	29
CTACGCGtATCTCTATATTTCGG	0.14%	27

***OsPDS*-sgRNA01**

Protospacer PAM

GTTGGTCTTTGCTCCTGCAGAGG	Ratio	Reads
GTTGGTCTTTGCTCCTGCAGAGG	77.30%	10288
GTTGGTtTTTGCTCCTGCAGAGG	20.32%	2705
GTTGGTCTTTGCTCCTGCAGAGG	0.07%	9
GTTGGTCTTTGCTCCTGCAGAGG	0.07%	9
GTTGGTCTTTGCTCCTGCAGAGG	0.06%	8
GTTGGTCTTTGCTCCTGCAGAGG	0.05%	7
GTTGGTCTTTGCTCCTGCAGAGG	0.05%	7
GTTGGTCTTTGCTCCTGCAGAGG	0.05%	7
GTTGGTCTTTGCTCCTGCAGAGG	0.04%	5
GTTGGTCTTTGCTCCTGCAGAGG	0.04%	5

***OsSPL14*-sgRNA01**

Protospacer PAM

TCTTCTGTCAACCCAGCCATGGG	Ratio	Reads
TCTTCTGTCAACCCAGCCATGGG	87.00%	40433
TCTTtTGTCAACCCAGCCATGGG	9.54%	4435
TCTTtTGTtAACCCAGCCATGGG	0.32%	150
TCTTCTGTtAACCCAGCCATGGG	0.22%	102
TCTTCTGTCAACtCAGCCATGGG	0.09%	40
TCTTtTGTCgACCCAGCCATGGG	0.08%	38
TCTTCTGTCAAtCCAGCCATGGG	0.05%	23
TCTTCTGTCAACCCAGCCATGGG	0.05%	21
TCTTCTGTCAACCCAGCCATGGG	0.04%	18
TCTTtTGTCAACtCAGCCATGGG	0.03%	16

Supplementary Figure 11. Genotyping data of the most frequent reads at the 12 target sites in rice protoplasts treated with TadDE. Sequences in lowercase red indicate base editing outcomes. The values on the right represent the ratio and reads of mutation alleles.

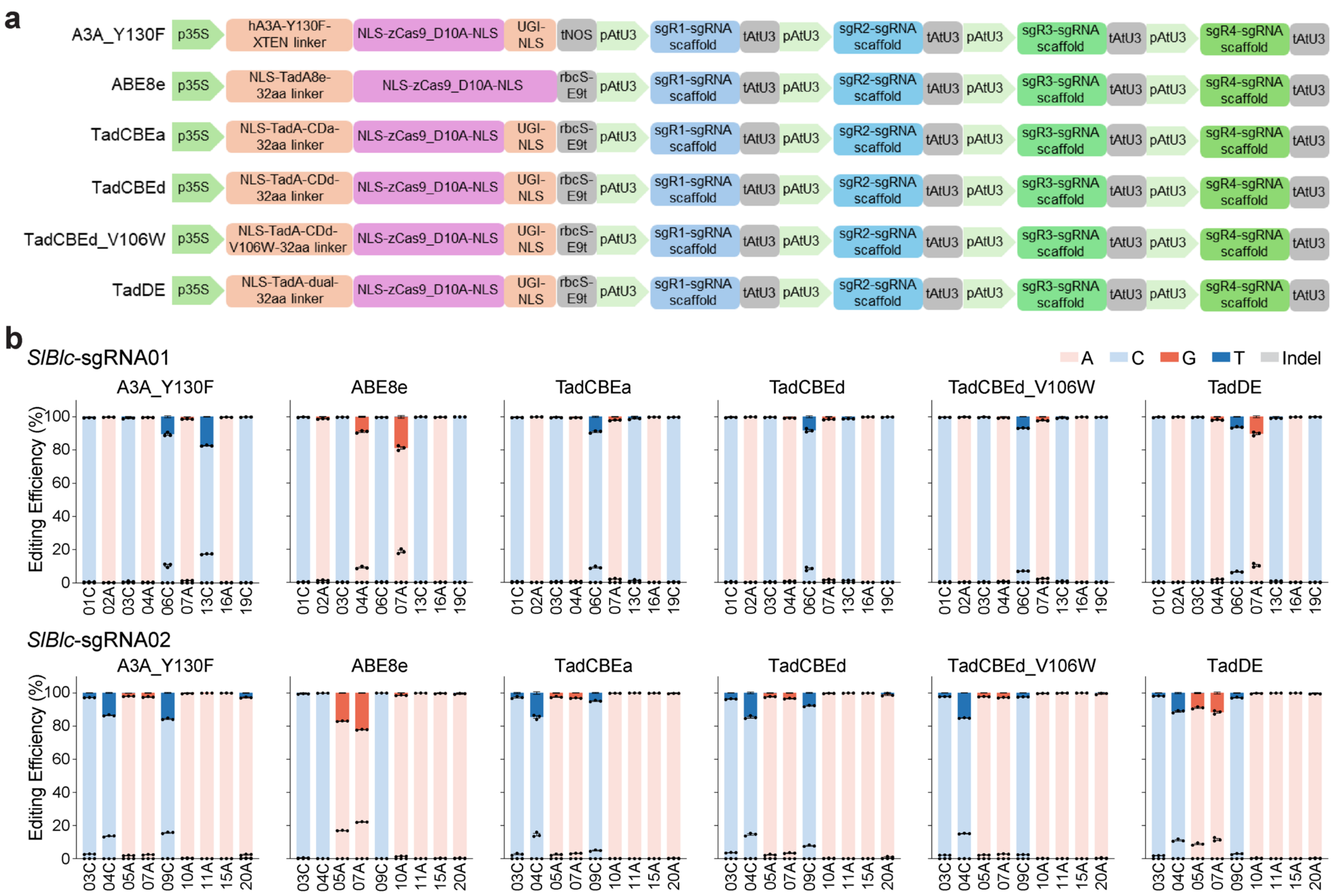

Supplementary Figure 12. Assessment of four TadA-8e derived cytosine and dual base editors in tomato protoplasts at four targets. (a) Base editor constructs in tomato. The deaminase and nCas9 proteins are driven by the 35S promoter, and the sgRNA expression cassette is initiated by the AtU6 promoter. **(b)** Bar plots showing the on-target DNA base editing frequencies and window of cytosines with C-to-T edits and adenines with A-to-G edits at 2 target sites in tomato protoplasts. Each Bar represents the average of three independent replicates (n = 3) at each base. Data are presented as mean values ± SEM.

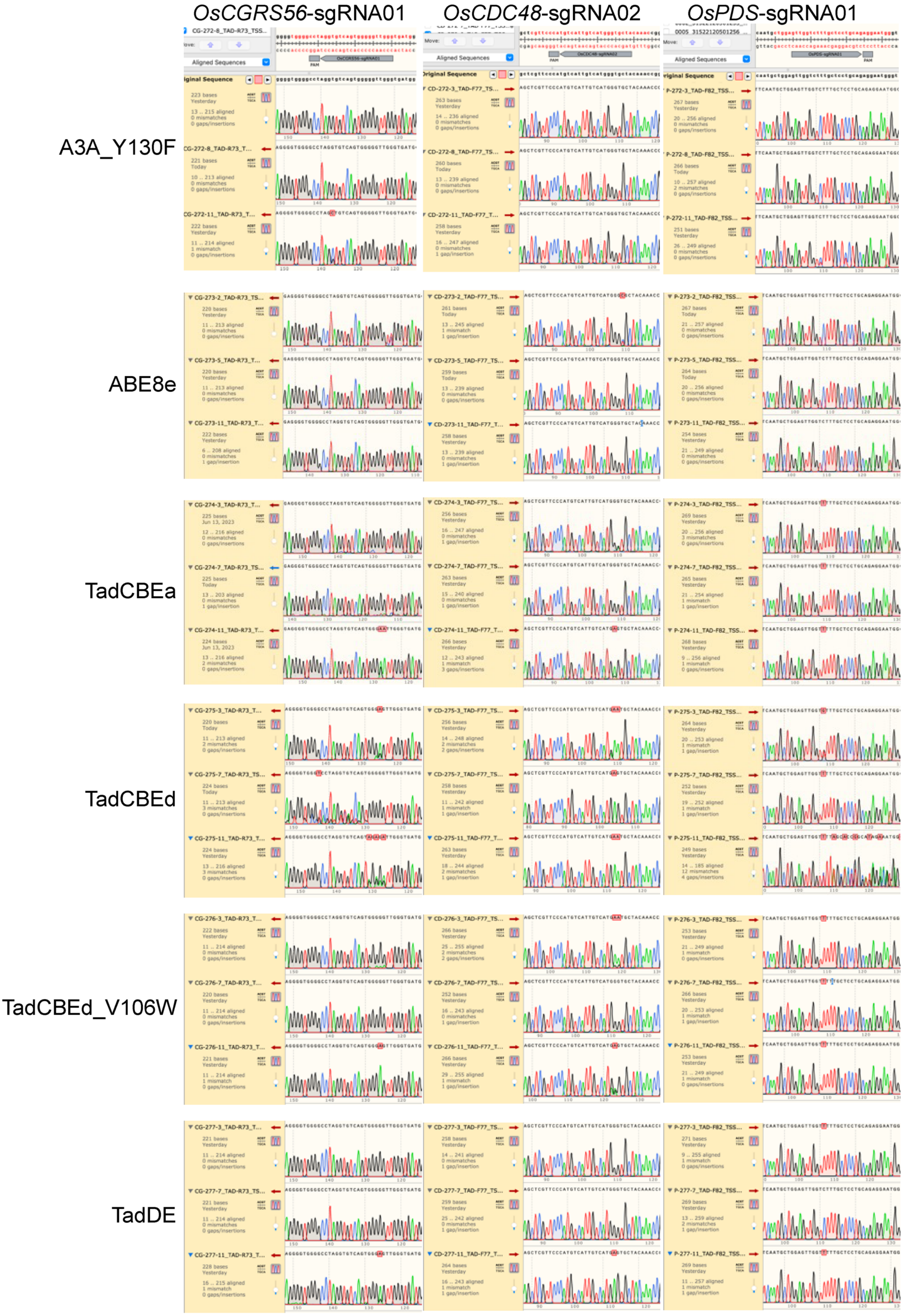

Supplementary Figure 13. Sanger sequencing to confirm base editing events for subsequent WGS analysis. Three randomly selected transgenic positive rice lines were chosen for A3A_Y130F, ABE8e, TadCBEa, TadCBEd, TadCBEd_V106W, and TadDE, and the editing status of three target sites was initially assessed through Sanger sequencing.

OsCGRS56-sgRNA01

```
                  Protospacer            PAM
Reference     CCCAACCCCCACTGACACCTAGG
TadCBEa_02    CCCAACtCCCACTGACACCTAGG
TadCBEa_07    CCCAAttCCCACTGACACCTAGG
TadCBEa_07    CCCAAtCCCCACTGACACCTAGG
TadCBEa_08    CCCAACtCCCACTGACACCTAGG
TadCBEa_12    CCCAACtCCCACTGACACCTAGG
TadCBEa_15    CCCAACtCCCACTGACACCTAGG
```

OsAAT-sgRNA02

```
                  Protospacer            PAM
Reference     CTGCGCCCCGCGCACGTTAGCGG
TadCBEa_02    CTGCGCttCGCGCACGTTAGCGG
TadCBEa_02    CTGCGtCtCGCGCACGTTAGCGG
TadCBEa_03    CTGCGCttCGCGCACGTTAGCGG
TadCBEa_05    CTGCGtCtCGCGCACGTTAGCGG
TadCBEa_07    CTGCGtCtCGCGCACGTTAGCGG
TadCBEa_07    CTGtGCCtCGCGCACGTTAGCGG
TadCBEa_08    CTGCGtttCGCGCACGTTAGCGG
TadCBEa_09    CTGCGttCCGCGCACGTTAGCGG
TadCBEa_10    CTGCGCttCGCGCACGTTAGCGG
TadCBEa_11    CTGCGCCtCGCGCACGTTAGCGG
TadCBEa_12    CTGCGCttCGCGCACGTTAGCGG
TadCBEa_12    CTGCGtCtCGCGCACGTTAGCGG
TadCBEa_13    CTGCGCttCGCGCACGTTAGCGG
TadCBEa_14    CTGCGCttCGCGCACGTTAGCGG
TadCBEa_14    CTGCGttCCGCGCACGTTAGCGG
TadCBEa_15    CTGCGtCtCGCGCACGTTAGCGG
TadCBEa_15    CTGCGtttCGCGCACGTTAGCGG
TadCBEa_17    CTGCGCttCGCGCACGTTAGCGG
TadCBEa_18    CTGCGCttCGCGCACGTTAGCGG
```

OsACC-sgRNA02

```
                  Protospacer            PAM
Reference     TCCACAGCTATCACACCCACTGG
TadCBEa_02    TCCACgGCTATCACACCCACTGG
TadCBEa_09    TCCAtAGCTATCACACCCACTGG
TadCBEa_10    TCCAtAGCTATCACACCCACTGG
TadCBEa_12    TCCACgGCTATCACACCCACTGG
TadCBEa_12    TCCAtAGCTATCACACCCACTGG
TadCBEa_15    TCCAtAGCTATCACACCCACTGG
```

OsALS-sgRNA02

```
                  Protospacer            PAM
Reference     TCTCCATGGACGCGCCGCCCGGG
TadCBEa_02    TCTCtATGGACGCGCCGCCCGGG
TadCBEa_02    TCTCtgTGGACGCGCCGCCCGGG
TadCBEa_03    TCTCtATGGACGCGCCGCCCGGG
TadCBEa_07    TCTCtATGGACGCGCCGCCCGGG
TadCBEa_10    TCTCtATGGACGCGCCGCCCGGG
TadCBEa_11    TCTCtATGGACGCGCCGCCCGGG
TadCBEa_12    TCTCtATGGACGCGCCGCCCGGG
TadCBEa_12    TCTCtgTGGACGCGCCGCCCGGG
TadCBEa_13    TCTCtATGGACGCGCCGCCCGGG
TadCBEa_14    TCTCtATGGACGCGCCGCCCGGG
TadCBEa_15    TCTCtATGGACGCGCCGCCCGGG
TadCBEa_18    TCTCtATGGACGCGCCGCCCGGG
```

OsCDC48-sgRNA02

```
                  Protospacer            PAM
Reference     TAGCACCCATGACAATGACATGG
TadCBEa_02    TAGCAtCCATGACAATGACATGG
TadCBEa_02    TAGCACtCATGACAATGACATGG
TadCBEa_03    TAGCAtCCATGACAATGACATGG
TadCBEa_05    TAGCAtCCATGACAATGACATGG
TadCBEa_07    TAGCACtCATGACAATGACATGG
TadCBEa_09    TAGCgCtCATGACAATGACATGG
TadCBEa_10    TAGCACtCATGACAATGACATGG
TadCBEa_10    TAGCACtCATGACAATGACATGG
TadCBEa_11    TAGCACtCATGACAATGACATGG
TadCBEa_12    TAGCAttCATGACAATGACATGG
TadCBEa_12    TAGCACtCATGACAATGACATGG
TadCBEa_12    TAGCAtCCATGACAATGACATGG
TadCBEa_14    TAGCACtCATGACAATGACATGG
TadCBEa_14    TAGtACtCATGACAATGACATGG
TadCBEa_15    TAGCAttCATGACAATGACATGG
TadCBEa_15    TAGCACttATGACAATGACATGG
TadCBEa_17    TAGCACtCATGACAATGACATGG
TadCBEa_17    TAGCACttATGACAATGACATGG
TadCBEa_18    TAGCACtCATGACAATGACATGG
```

OsDEP1-sgRNA02

```
                  Protospacer            PAM
Reference     AGCACATGAGAGAACAATATTGG
TadCBEa_02    AGCAtATGAGAGAACAATATTGG
TadCBEa_03    AGCAtATGAGAGAACAATATTGG
TadCBEa_05    AGCAtATGAGAGAACAATATTGG
TadCBEa_07    AGCAtATGAGAGAACAATATTGG
TadCBEa_12    AGCAtgTGAGAGAACAATATTGG
TadCBEa_12    AGCAtATGAGAGAACAATATTGG
TadCBEa_14    AGCAtATGAGAGAACAATATTGG
TadCBEa_14    AGCAtgTGAGAGAACAATATTGG
TadCBEa_15    AGCAtATGAGAGAACAATATTGG
TadCBEa_17    AGCAtATGAGAGAACAATATTGG
```

OsGS3-sgRNA02

```
                  Protospacer            PAM
Reference     CTACGCGCATCTCTATATTTCGG
TadCBEa_02    CTACGCGCATtTCTATATTTCGG
TadCBEa_02    CTAtGCGCATCTCTATATTTCGG
TadCBEa_03    CTACGCGCATtTCTATATTTCGG
TadCBEa_03    CTAtGtGCATCTCTATATTTCGG
TadCBEa_05    CTACGtGCATCTCTATATTTCGG
TadCBEa_07    CTACGtGCATCTCTATATTTCGG
TadCBEa_07    CTACGtGCATtTCTATATTTCGG
TadCBEa_09    CTAtGtGCATCTCTATATTTCGG
TadCBEa_10    CTACGtGCATCTCTATATTTCGG
TadCBEa_10    CTAtGtGCATCTCTATATTTCGG
TadCBEa_11    CTAtGtGCATCTCTATATTTCGG
TadCBEa_12    CTACGtGCATCTCTATATTTCGG
TadCBEa_12    CTACGCGCATtTCTATATTTCGG
TadCBEa_14    CTACGtGCATCTCTATATTTCGG
TadCBEa_15    CTACGtGCATCTCTATATTTCGG
TadCBEa_15    CTACGtGtATCTCTATATTTCGG
TadCBEa_17    CTACGtGCATCTCTATATTTCGG
TadCBEa_18    CTAtGtGCATCTCTATATTTCGG
```

OsLDMAR-sgRNA01

```
                  Protospacer            PAM
Reference     GTGCATCCTGGGAAAGCTTGTGG
TadCBEa_02    GTGtATtCTGGGAAAGCTTGTGG
TadCBEa_02    GTGCATttTGGGAAAGCTTGTGG
TadCBEa_03    GTGCATttTGGGAAAGCTTGTGG
TadCBEa_05    GTGCATtCTGGGAAAGCTTGTGG
TadCBEa_05    GTGtATtCTGGGAAAGCTTGTGG
TadCBEa_07    GTGCATttTGGGAAAGCTTGTGG
TadCBEa_08    GTGCATtCTGGGAAAGCTTGTGG
TadCBEa_09    GTGCATttTGGGAAAGCTTGTGG
TadCBEa_10    GTGCATtCTGGGAAAGCTTGTGG
TadCBEa_11    GTGCATttTGGGAAAGCTTGTGG
TadCBEa_12    GTGtATtCTGGGAAAGCTTGTGG
TadCBEa_12    GTGCATttTGGGAAAGCTTGTGG
TadCBEa_14    GTGCATttTGGGAAAGCTTGTGG
TadCBEa_14    GTGtATtCTGGGAAAGCTTGTGG
TadCBEa_15    GTGCATttTGGGAAAGCTTGTGG
TadCBEa_17    GTGCATttTGGGAAAGCTTGTGG
TadCBEa_18    GTGtATtCTGGGAAAGCTTGTGG
TadCBEa_18    GTGCATttTGGGAAAGCTTGTGG
```

OsPDS-sgRNA01

```
                  Protospacer            PAM
Reference     GTTGGTCTTTGCTCCTGCAGAGG
TadCBEa_02    GTTGGTtTTTGCTCCTGCAGAGG
TadCBEa_03    GTTGGTtTTTGCTCCTGCAGAGG
TadCBEa_05    GTTGGTtTTTGCTCCTGCAGAGG
TadCBEa_07    GTTGGTtTTTGCTCCTGCAGAGG
TadCBEa_08    GTTGGTtTTTGCTCCTGCAGAGG
TadCBEa_08    GTTGGTtTTTGCTCCTGCAGAGG
TadCBEa_09    GTTGGTtTTTGCTCCTGCAGAGG
TadCBEa_10    GTTGGTtTTTGCTCCTGCAGAGG
TadCBEa_11    GTTGGTtTTTGCTCCTGCAGAGG
TadCBEa_12    GTTGGTtTTTGCTCCTGCAGAGG
TadCBEa_14    GTTGGTtTTTGCTCCTGCAGAGG
TadCBEa_15    GTTGGTtTTTGCTCCTGCAGAGG
TadCBEa_17    GTTGGTtTTTGCTCCTGCAGAGG
TadCBEa_18    GTTGGTtTTTGCTCCTGCAGAGG
```

OsSPL14-sgRNA01

```
                  Protospacer            PAM
Reference     TCTTCTGTCAACCCAGCCATGGG
TadCBEa_02    TCTTtTGTCAACCCAGCCATGGG
TadCBEa_03    TCTTtTGTCAACCCAGCCATGGG
TadCBEa_05    TCTTtTGTCAACCCAGCCATGGG
TadCBEa_07    TCTTtTGTCAACCCAGCCATGGG
TadCBEa_08    TCTTtTGTCAACCCAGCCATGGG
TadCBEa_09    TCTTtTGTCAACCCAGCCATGGG
TadCBEa_10    TCTTtTGTCAACCCAGCCATGGG
TadCBEa_11    TCTTtTGTCAACCCAGCCATGGG
TadCBEa_12    TCTTtTGTCAACCCAGCCATGGG
TadCBEa_13    TCTTtTGTCAACCCAGCCATGGG
TadCBEa_14    TCTTtTGTCAACCCAGCCATGGG
TadCBEa_15    TCTTtTGTCAACCCAGCCATGGG
TadCBEa_17    TCTTtTGTCAACCCAGCCATGGG
TadCBEa_18    TCTTtTGTCAACCCAGCCATGGG
```

Supplementary Figure 14. WGS genotyping data of multiplexed base editing T0 rice lines edited by TadCBEa. Genotypes of the transgenic plants at the target sites were obtained through WGS analysis. In the results, lowercase red letters represent mutated bases in the genome.

***OsCGRS56*-sgRNA01**

```
                     Protospacer      PAM
Reference  CCCAACCCCCACTGACACCTAGG
TadDE_02   CCCAAtCCCCACTGACACCTAGG
TadDE_02   CCCAACtCCCACTGACACCTAGG
TadDE_04   CCtAACCCCCACTGACACCTAGG
TadDE_06   CCtAACCCCCACTGACACCTAGG
TadDE_09   CCCAACtCCCACTGACACCTAGG
TadDE_09   CCCAAtCCCCACTGACACCTAGG
TadDE_11   CCCAACtCCCACTGACACCTAGG
TadDE_12   CCCAACtCCCACTGACACCTAGG
TadDE_14   CCCAAtCCCCACTGACACCTAGG
```

***OsAAT*-sgRNA02**

```
                     Protospacer      PAM
Reference  CTGCGCCCCGCGCACGTTAGCGG
TadDE_02   CTGCGtttCGCGCACGTTAGCGG
TadDE_02   CTGCGtCtCGCGCACGTTAGCGG
TadDE_04   CTGCGtCtCGCGCACGTTAGCGG
TadDE_06   CTGCGtCtCGCGCACGTTAGCGG
TadDE_09   CTGCGtCtCGCGCACGTTAGCGG
TadDE_10   CTGCGtCtCGCGCACGTTAGCGG
TadDE_10   CTGCGCttCGCGCACGTTAGCGG
TadDE_11   CTGCGtCtCGCGCACGTTAGCGG
TadDE_12   CTGCGCtttGCGCACGTTAGCGG
TadDE_12   CTGCGCCtCGCGCACGTTAGCGG
TadDE_13   CTGCGCttCGCGCACGTTAGCGG
TadDE_14   CTGCGtCtCGCGCACGTTAGCGG
TadDE_15   CTGtGCCCCGCGCACGTTAGCGG
```

***OsALS*-sgRNA02**

```
                     Protospacer      PAM
Reference  TCTCCATGGACGCGCCGCCCGGG
TadDE_02   TCTCtATGGACGCGCCGCCCGGG
TadDE_04   TCTCtATGGACGCGCCGCCCGGG
TadDE_09   TCTCtATGGACGCGCCGCCCGGG
TadDE_10   TCTCtATGGACGCGCCGCCCGGG
TadDE_11   TCTCtATGGACGCGCCGCCCGGG
TadDE_12   TCTCtATGGACGCGCCGCCCGGG
TadDE_13   TCTCtATGGACGCGCCGCCCGGG
TadDE_14   TCTCtATGGACGCGCCGCCCGGG
```

***OsACC*-sgRNA02**

```
                     Protospacer      PAM
Reference  TCCACAGCTATCACACCCACTGG
TadDE_12   TCCAtAGCTATCACACCCACTGG
```

***OsCDC48*-sgRNA02**

```
                     Protospacer      PAM
Reference  TAGCACCCATGACAATGACATGG
TadDE_02   TAGCACtCATGACAATGACATGG
TadDE_02   TAGCACCtATGACAATGACATGG
TadDE_04   TAGCACtCATGACAATGACATGG
TadDE_04   TAGCACtCATGACAATGACATGG
TadDE_09   TAGCACtCATGACAATGACATGG
TadDE_11   TAGCACtCATGACAATGACATGG
TadDE_12   TAGCACtCATGACAATGACATGG
TadDE_13   TAGCACtCATGACAATGACATGG
TadDE_13   TAGCAtCCATGACAATGACATGG
TadDE_14   TAGCACtCATGACAATGACATGG
```

***OsDEP1*-sgRNA02**

```
                     Protospacer      PAM
Reference  AGCACATGAGAGAACAATATTGG
TadDE_02   AGtACATGAGAGAACAATATTGG
TadDE_04   AGCAtATGAGAGAACAATATTGG
TadDE_06   AGCAtATGAGAGAACAATATTGG
TadDE_09   AGCAtATGAGAGAACAATATTGG
TadDE_12   AGCAtATGAGAGAACAATATTGG
TadDE_13   AGtACATGAGAGAACAATATTGG
```

***OsSPL14*-sgRNA01**

```
                     Protospacer      PAM
Reference  TCTTCTGTCAACCCAGCCATGGG
TadDE_02   TCTTtTGTCAACCCAGCCATGGG
TadDE_04   TCTTtTGTCAACCCAGCCATGGG
TadDE_09   TCTTtTGTCAACCCAGCCATGGG
TadDE_10   TCTTtTGTCAACCCAGCCATGGG
TadDE_11   TCTTtTGTCAACCCAGCCATGGG
TadDE_12   TCTTtTGTCAACCCAGCCATGGG
TadDE_13   TCTTtTGTCAACCCAGCCATGGG
TadDE_14   TCTTtTGTCAACCCAGCCATGGG
```

***OsLDMAR*-sgRNA01**

```
                     Protospacer      PAM
Reference  GTGCATCCTGGGAAAGCTTGTGG
TadDE_02   GTGCATttTGGGAAAGCTTGTGG
TadDE_03   GTGCATtCTGGGAAAGCTTGTGG
TadDE_04   GTGCATttTGGGAAAGCTTGTGG
TadDE_04   GTGtATttTGGGAAAGCTTGTGG
TadDE_06   GTGCATtCTGGGAAAGCTTGTGG
TadDE_09   GTGtATtCTGGGAAAGCTTGTGG
TadDE_09   GTGCATtCTGGGAAAGCTTGTGG
TadDE_10   GTGtATtCTGGGAAAGCTTGTGG
TadDE_11   GTGCATttTGGGAAAGCTTGTGG
TadDE_12   GTGCATtCTGGGAAAGCTTGTGG
TadDE_13   GTGCATttTGGGAAAGCTTGTGG
TadDE_14   GTGCATttTGGGAAAGCTTGTGG
```

***OsPDS*-sgRNA01**

```
                     Protospacer      PAM
Reference  GTTGGTCTTTGCTCCTGCAGAGG
TadDE_02   GTTGGTtTTTGCTCCTGCAGAGG
TadDE_03   GTTGGTtTTTGCTCCTGCAGAGG
TadDE_04   GTTGGTtTTTGCTCCTGCAGAGG
TadDE_06   GTTGGTtTTTGCTCCTGCAGAGG
TadDE_09   GTTGGTtTTTGCTCCTGCAGAGG
TadDE_10   GTTGGTtTTTGCTCCTGCAGAGG
TadDE_11   GTTGGTtTTTGCTCCTGCAGAGG
TadDE_12   GTTGGTtTTTGCTCCTGCAGAGG
TadDE_13   GTTGGTtTTTGCTCCTGCAGAGG
TadDE_14   GTTGGTtTTTGCTCCTGCAGAGG
TadDE_15   GTTGGTtTTTGCTCCTGCAGAGG
```

***OsGS3*-sgRNA02**

```
                     Protospacer      PAM
Reference  CTACGCGCATCTCTATATTTCGG
TadDE_02   CTACGCGCATtTCTATATTTCGG
TadDE_02   CTACGtGCATCTCTATATTTCGG
TadDE_04   CTACGtGCATCTCTATATTTCGG
TadDE_09   CTACGCGCATtTCTATATTTCGG
TadDE_11   CTACGtGCATCTCTATATTTCGG
TadDE_13   CTACGtGCATCTCTATATTTCGG
TadDE_14   CTACGtGCATCTCTATATTTCGG
```

Supplementary Figure 15. WGS genotyping data of multiplexed base editing T0 rice lines edited by TadDE. Genotypes of the transgenic plants at the target sites were obtained through WGS analysis. In the results, lowercase red letters represent mutated bases in the genome.

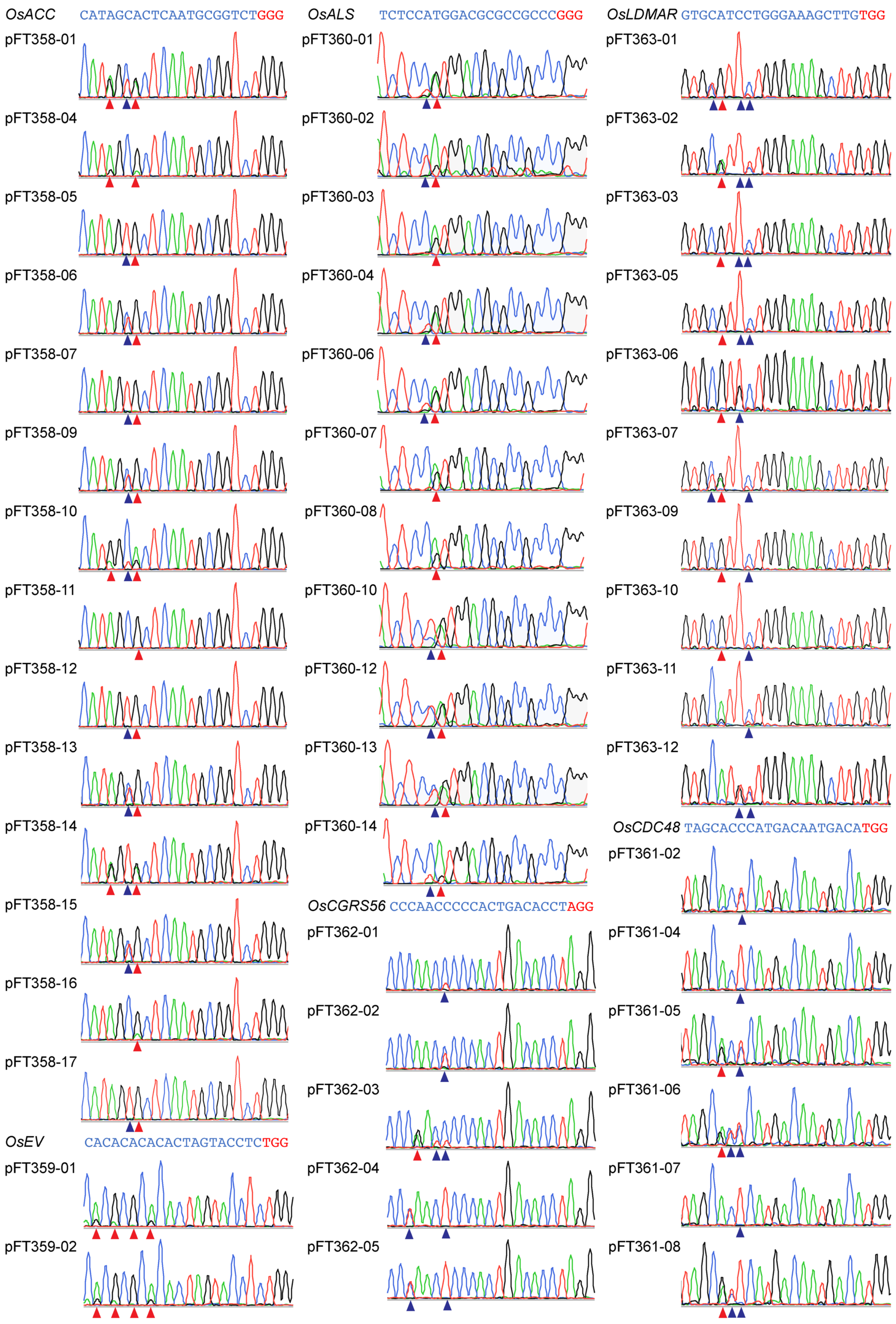

Supplementary Figure 16. Sanger sequencing genotyping results of singular base editing T0 rice lines edited by TadDE. The Sanger sequencing chromatogram of the target gene for singular site editing by TadDE. Blue arrows indicate C-to-T mutations, while red arrows indicate A-to-G mutations.

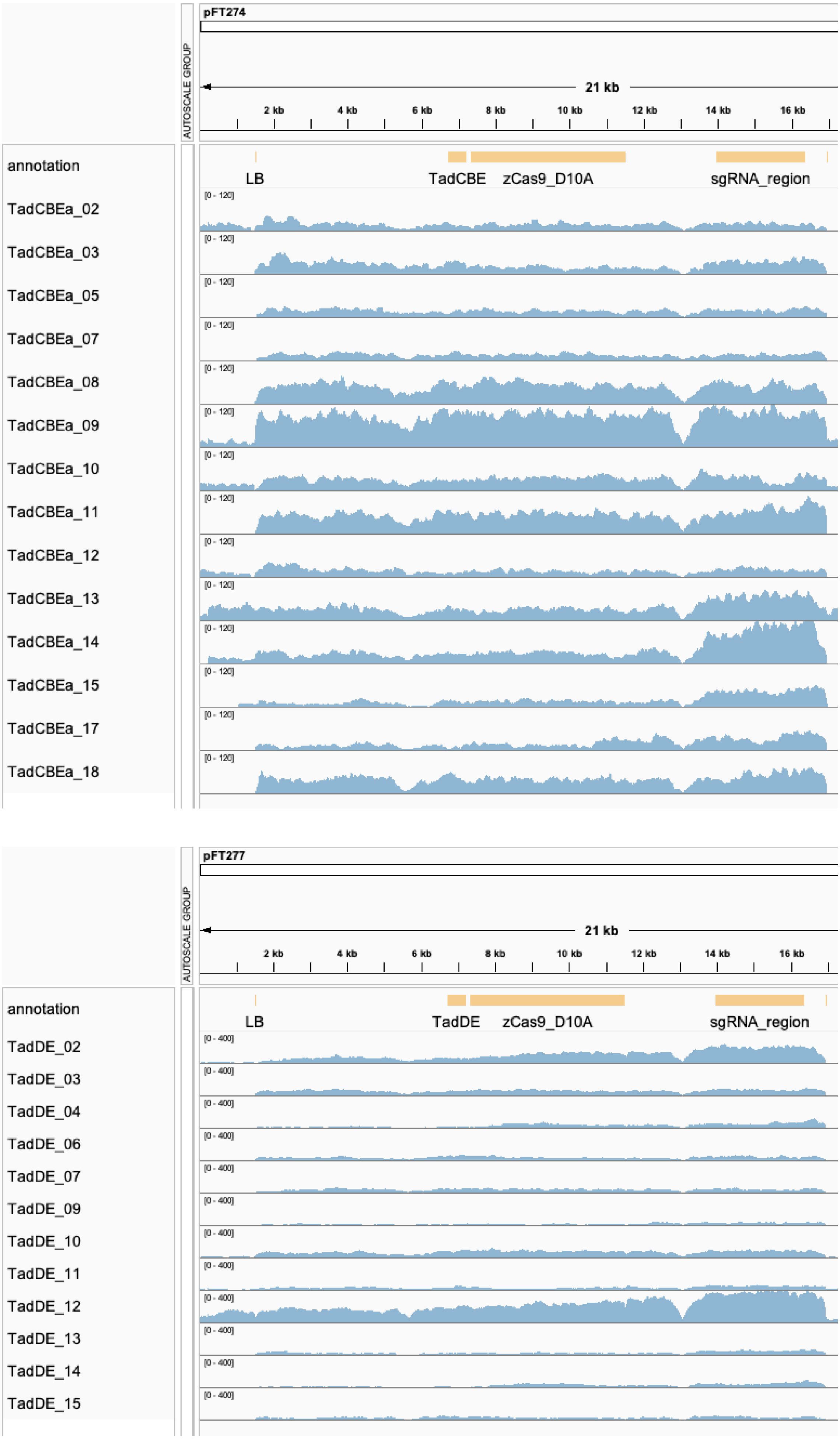

Supplementary Figure 17. Expression level of nCas9-deaminase protein and sgRNA determined by RNA-seq. The expression levels of the base editing vectors are presented for 14 individual TadCBEa plants and 12 individual TadDE plants.

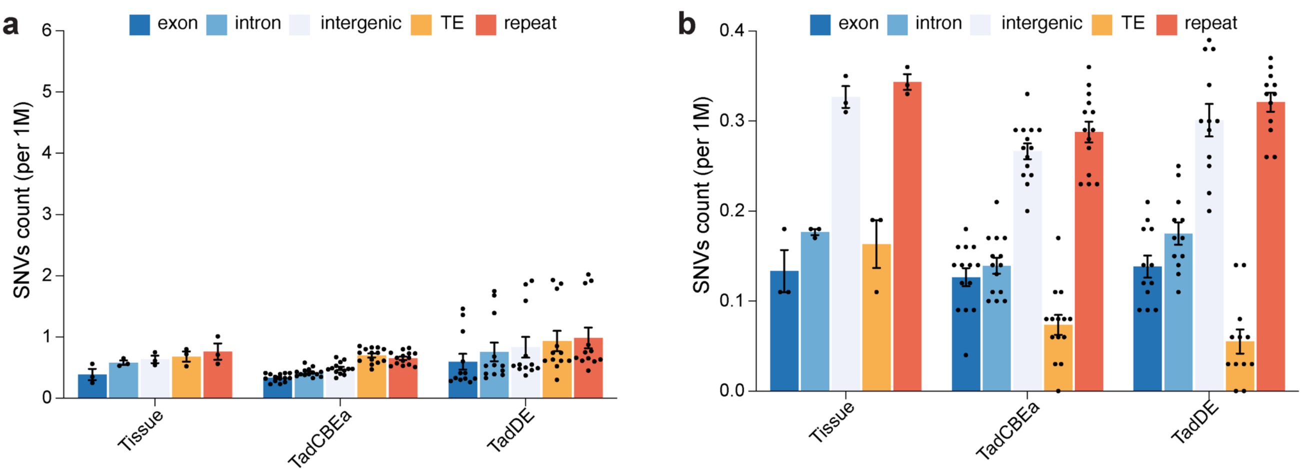

Supplementary Figure 18. Genomic annotation of detected both SNVs and InDels in WGS-analyzed rice T0 lines. (a) Ratio of SNVs in Tissue (n=3), TadCBEa (n=14), and TadDE (n=12) plants. Data are presented as mean values ± SEM. **(b)** Ratio of InDels in Tissue (n=3), TadCBEa (n=14), and TadDE (n=12) plants. Data are presented as mean values ± SEM.

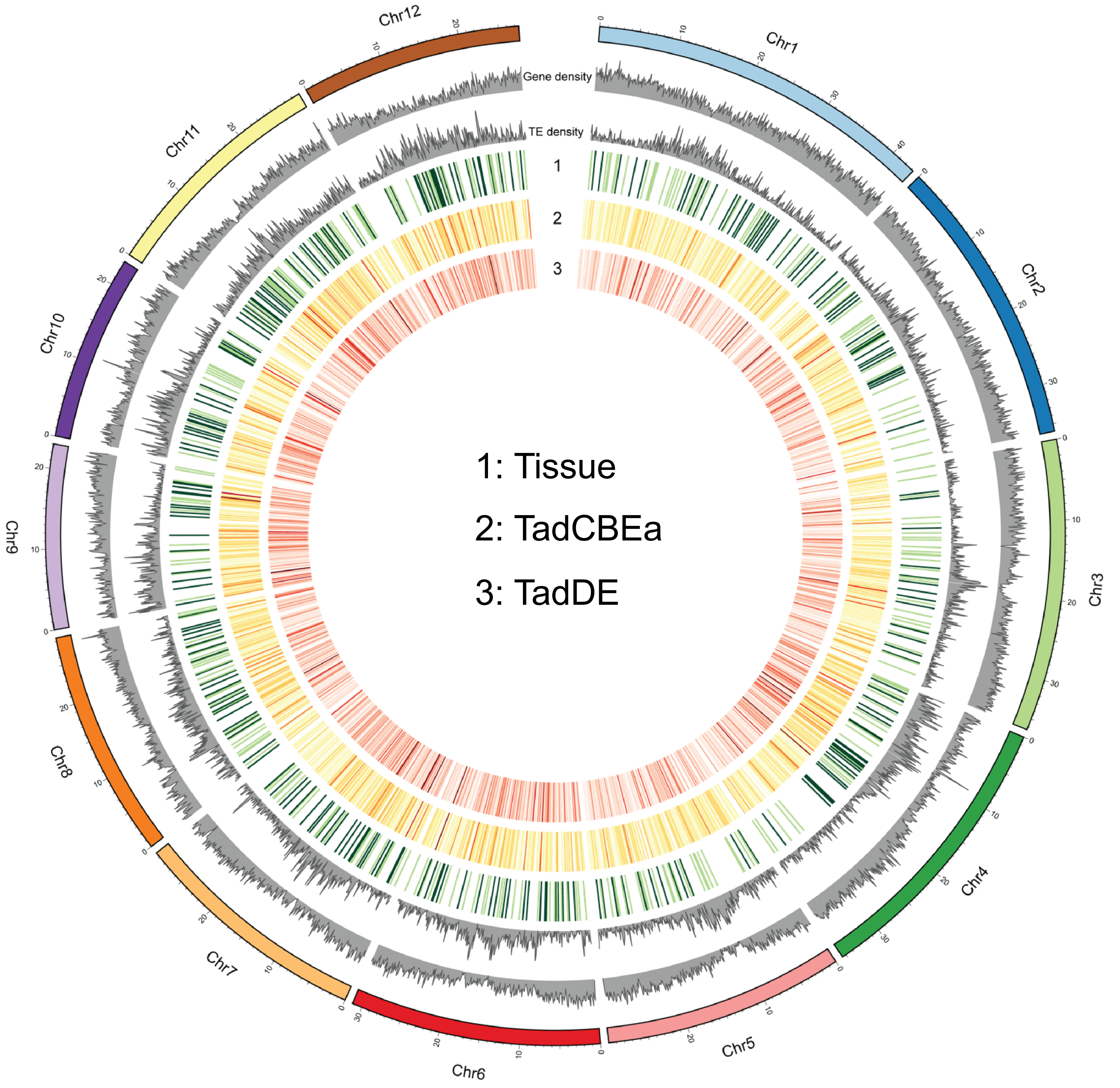

Supplementary Figure 19. Comparison of SNV numbers and distribution in the edited T0 rice plants by TadCBEa and TadDE across the entire rice genome.

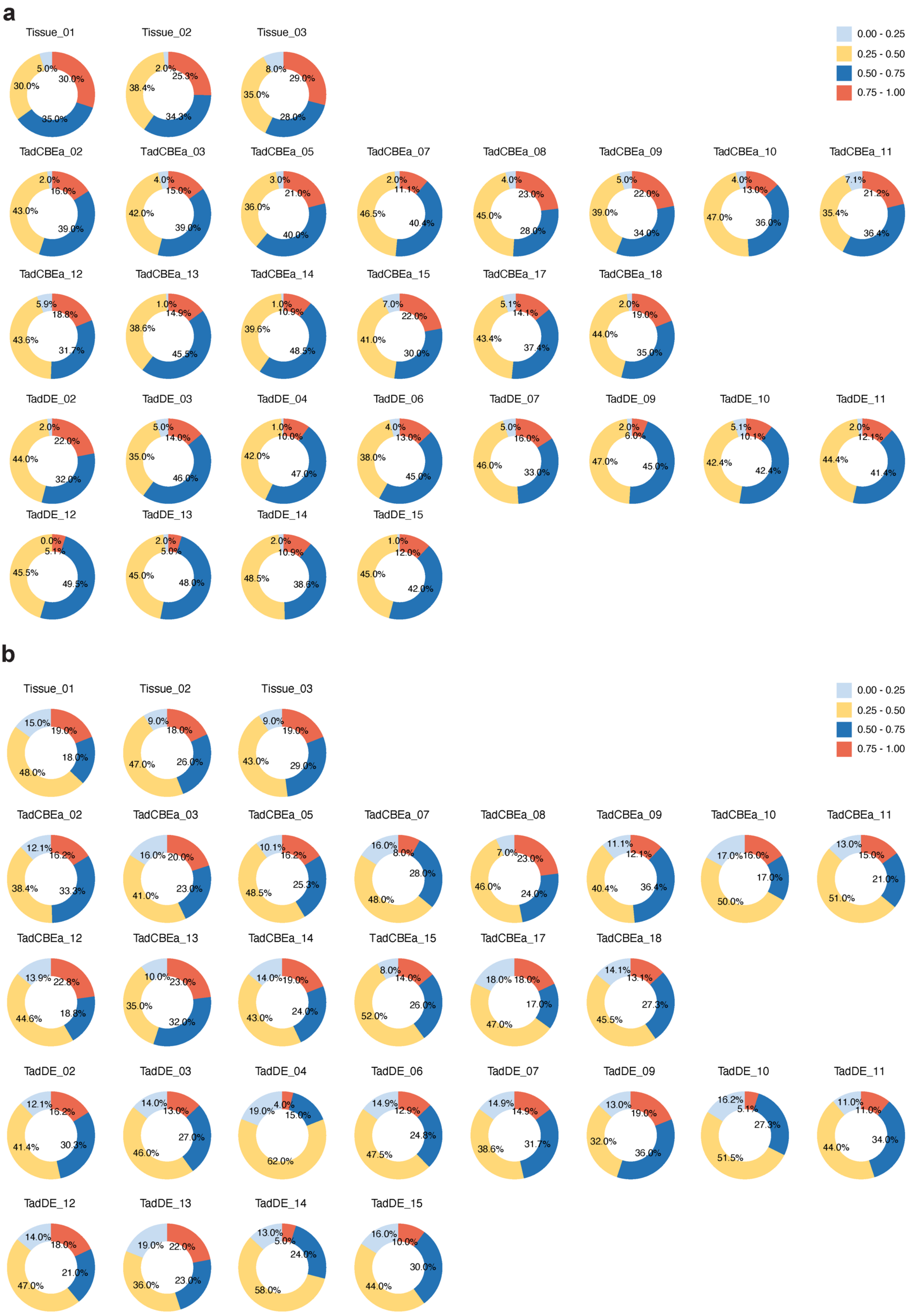

Supplementary Figure 20. Allele frequencies of SNVs (a) and InDels (b) among the edited T0 rice plants by TadCBEa and TadDE.

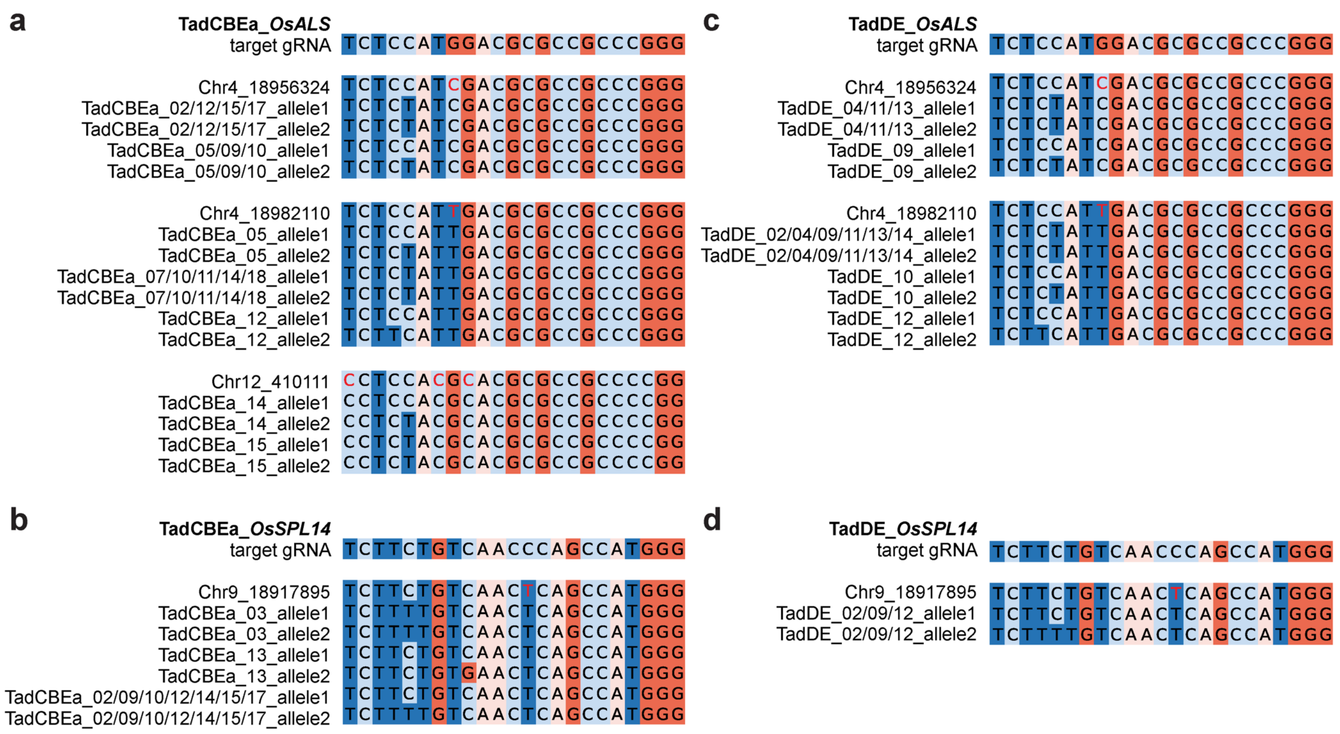

Supplementary Figure 21. Sequence analysis of potential off-target sites with mismatches ≤ 3 among 10 target sites in the TadCBEa and TadDE edited T0 rice plants. **(a)** Potential off-target sites of OsALS-sgRNA02 in TadCBEa. **(b)** Potential off-target sites of OsSPL14-sgRNA01 in TadCBEa. **(c)** Potential off-target sites of OsALS-sgRNA02 in TadDE. **(d)** Potential off-target sites of OsSPL14-sgRNA01 in TadDE.

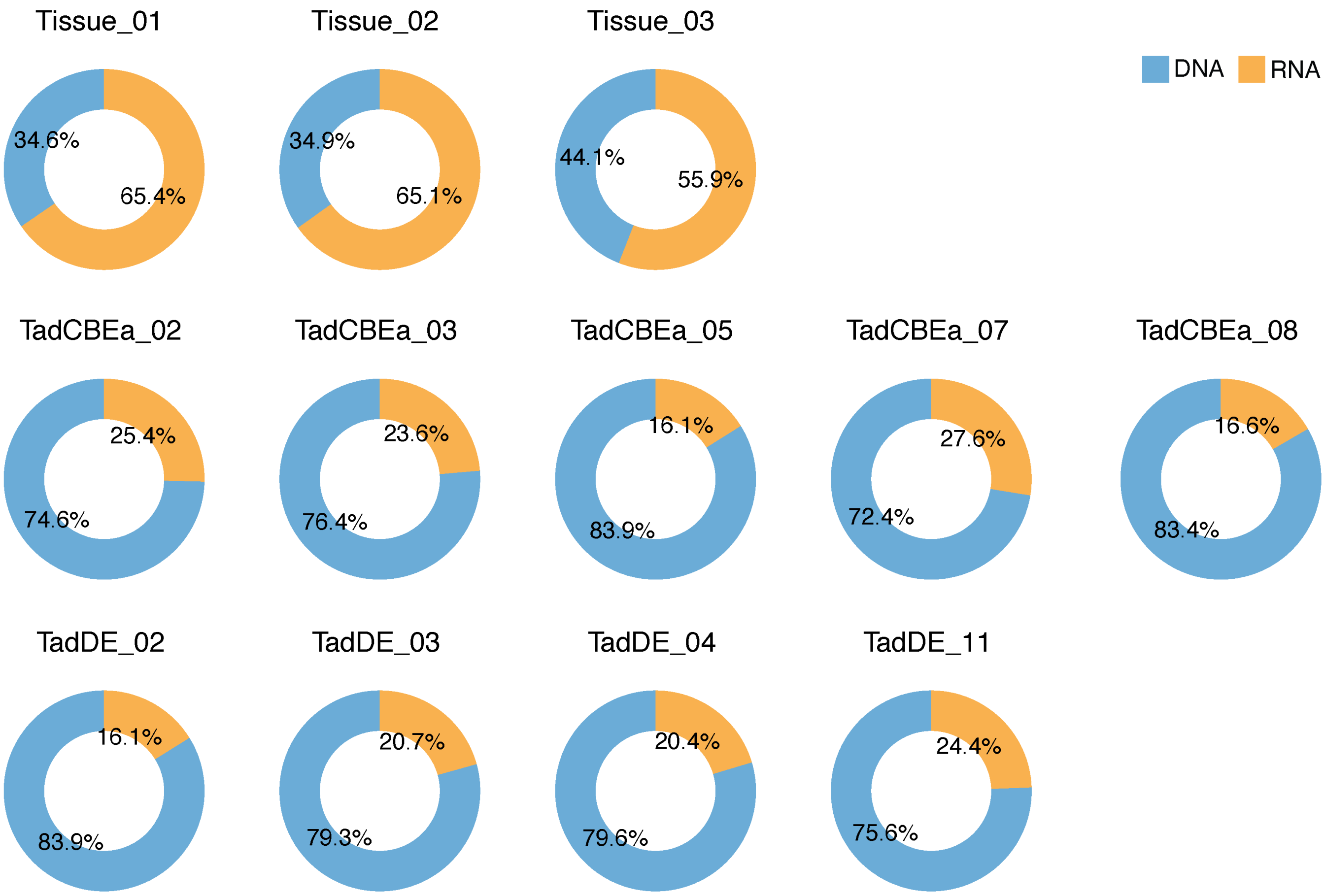

Supplementary Figure 22. Off-target assessment of base editing systems in rice by transcriptome sequencing. The identified RNA mutation sites are derived from the ratio of DNA to RNA levels. Sites with DNA mutation rates <5% but RNA mutation rates >5% are considered at the RNA level.

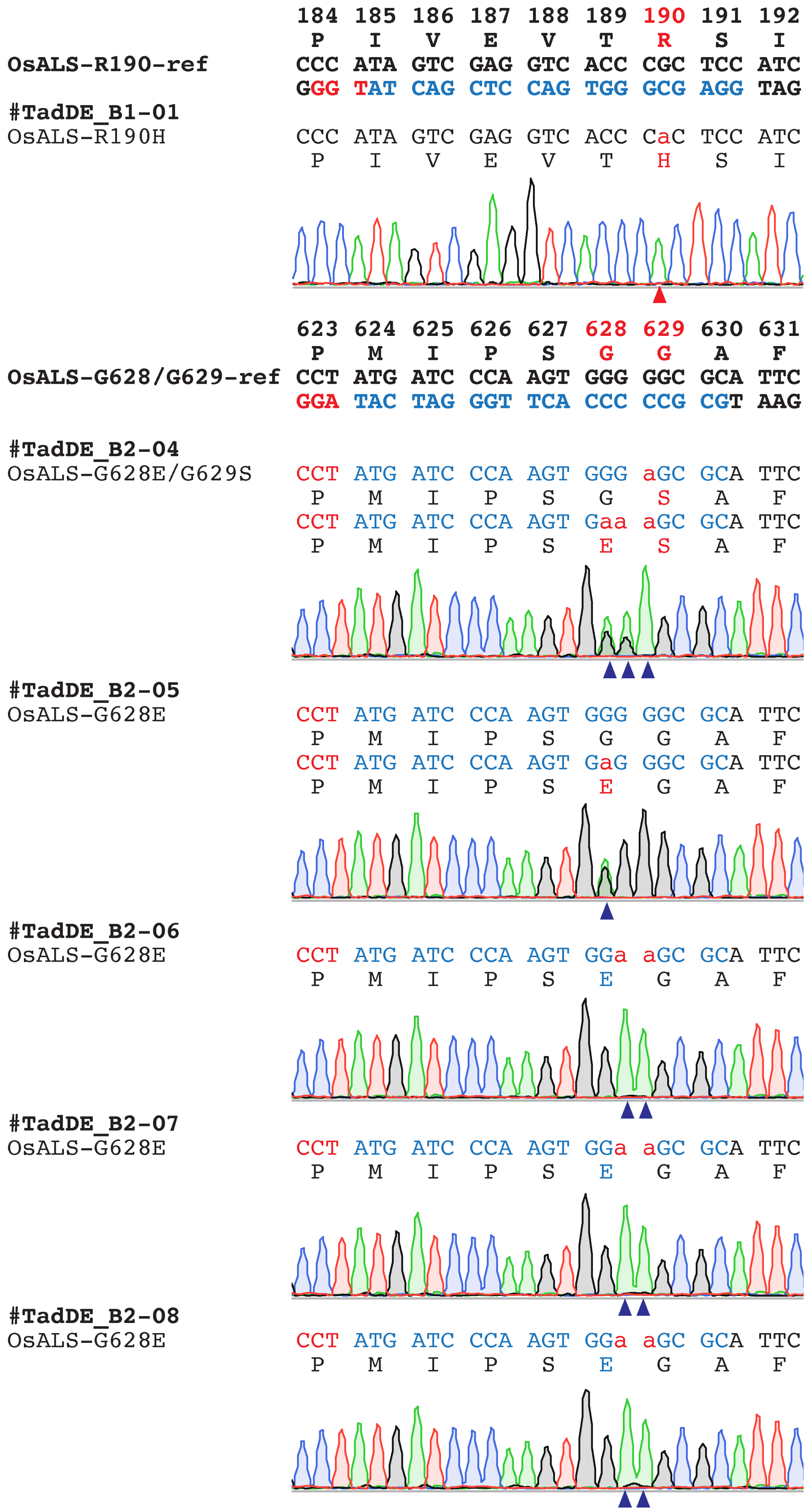

Supplementary Figure 23. Sanger sequencing results of *OsALS* PCR products in herbicide-resistant rice plants edited by TadDE.

***OsSPL14*-sgR05-ref** **5′-ACC GAC TCG AGC TGT GCT CTC TCT CTT CTG TCA-3′**
3′-TGG CTG AGC TCG ACA CGA GAG AGA GAA GAC AGT-5′

#TadDE_B4-12 allele01 ACC GAC TCG AGC TGT GCT CTC TCT CTT CTG TCA
allele02 ACC GAC TCG AGC TGT GCT CcC TCT CTT CTG TCA
T D S S C A P S L L S

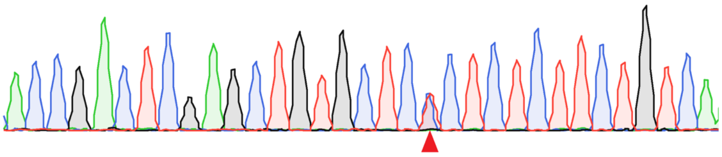

#TadDE_B4-13 allele01 ACC GAC TCG AGC TGT GCc CcC TCT CTT CTG TCA
allele02 ACC GAC TCG AGC TGT GCc CcC TCT CTT CTG TCA
T D S S C A P S L L S

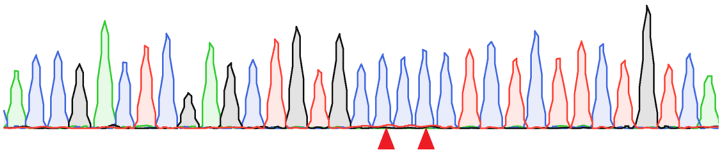

#TadDE_B4-16 allele01 ACC GAC TCG AGC TGT GCT CTC TCT CTT CTG TCA
allele02 ACC GAC TCG AGC TGT GCc CTC TCT CTT CTG TCA
T D S S C A L S L L S

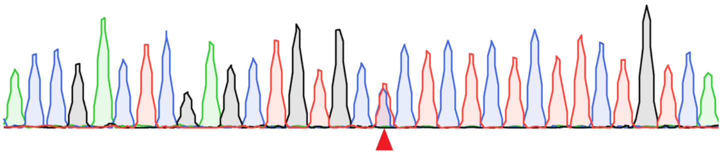

#TadDE_B4-18 allele01 ACC GAC TCG AGC TGT GCT CTC TCT CTT CTG TCA
allele02 ACC GAC TCG AGC TGc GCT CTC TCT CTT CTG TCA
T D S S C A L S L L S

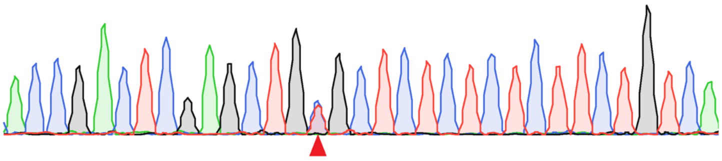

#TadDE_B4-21 allele01 ACC GAC TCG AGC TGT GCT CTC TCT CTT CTG TCA
allele02 ACC GAC TCG AGC TGT GCc CcC TCT CTT CTG TCA
T D S S C A P S L L S

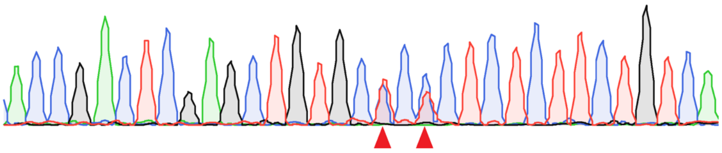

#TadDE_B4-24 allele01 ACC GAC TCG AGC TGc GCT CTC TCT CTT CTG TCA
allele02 ACC GAC TCG AGC TGc GCT CcC TCT CTT CTG TCA
T D S S C A P S L L S

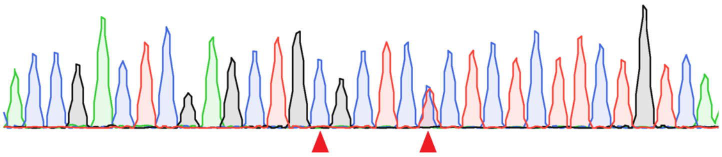

Supplementary Figure 24. Sanger sequencing results of *OsSPL14* PCR products in microRNA-resistant *OsSPL14* with synonymous mutations introduced by TadDE.

Supplementary Tables

Supplementary Table 1. T-DNA constructs used in this study.

Construct	Purpose
pTX1839	For A3A_Y130F-CBE base editing assay without sgRNA in rice
pTX1841	For ABE8e base editing assay without sgRNA in rice
pGEL850	For TadCBEa base editing assay without sgRNA in rice
pGEL851	For TadCBEd base editing assay without sgRNA in rice
pGEL852	For TadCBEd_V106W base editing assay without sgRNA in rice
pGEL853	For TadDE base editing assay without sgRNA in rice
pGEL854	For eTd-CBE base editing assay without sgRNA in rice
pGEL855	For Td-CBEmax base editing assay without sgRNA in rice
pGEL856	For TadDE-NG base editing assay without sgRNA in rice
pFT261	For A3A_Y130F-CBE base editing assay on multiplex array 01 in rice
pFT262	For ABE8e base editing assay on multiplex array 01 in rice
pFT263	For TadCBEa base editing assay on multiplex array 01 in rice
pFT264	For TadCBEd base editing assay on multiplex array 01 in rice
pFT265	For TadCBEd_V106W base editing assay on multiplex array 01 in rice
pFT266	For TadDE base editing assay on multiplex array 01 in rice
pFT267	For eTd-CBE base editing assay on multiplex array 01 in rice
pFT268	For Td-CBEmax base editing assay on multiplex array 01 in rice
pFT269	For TadDE-NG base editing assay on multiplex array 01 in rice
pFT272	For A3A_Y130F-CBE base editing assay on multiplex array 02 in rice
pFT273	For ABE8e base editing assay on multiplex array 02 in rice
pFT274	For TadCBEa base editing assay on multiplex array 02 in rice
pFT275	For TadCBEd base editing assay on multiplex array 02 in rice
pFT276	For TadCBEd_V106W base editing assay on multiplex array 02 in rice
pFT277	For TadDE base editing assay on multiplex array 02 in rice
pFT278	For eTd-CBE base editing assay on multiplex array 02 in rice
pFT279	For Td-CBEmax base editing assay on multiplex array 02 in rice
pFT280	For TadDE-NG base editing assay on multiplex array 02 in rice
pFT296	For TadDE base editing assay on OsALS-sgRNA04 in rice
pFT297	For TadDE base editing assay on OsALS-sgRNA06 in rice
pFT298	For TadDE base editing assay on OsALS-sgRNA17 in rice
pFT299	For TadDE base editing assay on OsALS-sgRNA18 in rice
pFT300	For TadDE base editing assay on OsALS-sgRNA19 in rice

Construct	Purpose
pFT301	For TadDE base editing assay on OsALS-sgRNA25 in rice
pFT304	For TadDE base editing assay on OsSPL14-sgRNA05 in rice
pLR5281	For A3A_Y130F base editing in tomato
pLR5282	For ABE8e base editing in tomato
pLR5284	For TadCBEa base editing in tomato
pLR5285	For TadCBEd base editing in tomato
pLR5286	For TadCBEd-V106W base editing in tomato
pLR5287	For TadDE base editing in tomato

Supplementary Table 2. Targets sites tested in this study.

Target site	Target gene	sgRNA sequence (5'-3')	PAM site	Target amino acid	Purpose
OsCGRS55-sgRNA01	OsCGRS55	CCACCCCTCCATCTCCTCCA	CGG	-	Multiplex array 01 base editing in rice
OsAAT-sgRNA01	OsAAT	CAAGGATCCCAGCCCCGTGA	AGG	-	Multiplex array 01 base editing in rice
OsACC-sgRNA01	OsACC	CATAGCACTCAATGCGGTCT	GGG	-	Multiplex array 01 base editing in rice
OsACTG-sgRNA01	OsACTG	ATCATCCGCCACGACGGCGG	CGG	-	Multiplex array 01 base editing in rice
OsALS-sgRNA01	OsALS	CGCGTCCATGGAGATCCACC	AGG	-	Multiplex array 01 base editing in rice
OsCDC48-sgRNA01	OsCDC48	GACCAGCCAGCGTCTGGCGC	CGG	-	Multiplex array 01 base editing in rice
OsD18-sgRNA01	OsD18	AGCAACCATTCATGGATGGA	TGG	-	Multiplex array 01 base editing in rice
OsDEP1-sgRNA01	OsDEP1	AGACAAGCTTGGCCCTCTTT	GGG	-	Multiplex array 01 base editing in rice
OsEV-sgRNA01	OsEV	CACACACACACTAGTACCTC	TGG	-	Multiplex array 01 base editing in rice
OsGRF1-sgRNA01	OsGRF1	GAACCGTTCAAGAAAGCCTG	TGG	-	Multiplex array 01 base editing in rice
OsCGRS56-sgRNA01	OsCGRS56	CCCAACCCCCACTGACACCT	AGG	-	Multiplex array 02 base editing in rice
OsAAT-sgRNA02	OsAAT	CTGCGCCCCGCGCACGTTAG	CGG	-	Multiplex array 02 base editing in rice
OsACC-sgRNA02	OsACC	TCCACAGCTATCACACCCAC	TGG	-	Multiplex array 02 base editing in rice
OsALS-sgRNA02	OsALS	TCTCCATGGACGCGCCGCCC	GGG	-	Multiplex array 02 base editing in rice
OsCDC48-sgRNA02	OsCDC48	TAGCACCCATGACAATGACA	TGG	-	Multiplex array 02 base editing in rice
OsDEP1-sgRNA02	OsDEP1	AGCACATGAGAGAACAATAT	TGG	-	Multiplex array 02 base editing in rice
OsGS3-sgRNA02	OsGS3	cTACGCGCATCTCTATATTT	CGG	-	Multiplex array 02 base editing in rice
OsLDMAR-sgRNA01	OsLDMAR	GTGCATCCTGGGAAAGCTTG	TGG	-	Multiplex array 02 base editing in rice
OsPDS-sgRNA01	OsPDS	GTTGGTCTTTGCTCCTGCAG	AGG	-	Multiplex array 02 base editing in rice
OsSPL14-sgRNA01	OsSPL14	TCTTCTGTCAACCCAGCCAT	GGG	-	Multiplex array 02 base editing in rice
OsALS-sgRNA04	OsALS	CAGGTCCCCCGCCGCATGAT	CGG	P171、R173	base editing in rice
OsALS-sgRNA06	OsALS	GCGGGGGACCTGGCCCGTGA	TGG	P171、R173	base editing in rice
OsALS-sgRNA17	OsALS	ggagcgggtgacctcgacta	TGG	R190H	base editing in rice
OsALS-sgRNA18	OsALS	GCGCCCCCACTTGGGATCAT	AGG	S627, G628E, G629S	base editing in rice
OsALS-sgRNA19	OsALS	cccaagtgggggcgcattca	AGG	S627, G628E, G629S	base editing in rice
OsALS-sgRNA25	OsALS	tggtggtgcaatgggaggat	AGG	W548L (prime editing)	base editing in rice
OsSPL14-sgRNA05	OsSPL14	agagagagcacagctcgagt	CGG	miRNA156 target region	base editing in rice
SIBlc-gR1	SolycBLC	CACAGCAGGTTTCGGAGGCT	GGG	-	Base editing in tomato
SiBlc-gR2	SolycBLC	GGCCATATCAATTTAGGGGA	CGG	-	Base editing in tomato
SolyA7-gR3	SolycAgo7	CAGCCTCCTAAGCTTAAACT	AGG	-	Base editing in tomato
SolyA7-gR4	SolycAgo7	AAGATCTCAGCGCGATGGTT	GGG	-	Base editing in tomato

Supplementary Table 3. The transformation efficiency of multiplexed genome base editing by TadA-8e derived TadCBEs and TadDE in rice T_0 lines.

No.	Vector	Editing type	Target site	No. of initial callus	No. of resistance callus from first round selection	No. of all regenerated T0 lines	No. of transgenic T0 lines
1	pFT261	A3A_Y130F	multiplex array 01	30	15	9	4
2	pFT262	ABE8e	multiplex array 01	60	13	12	1
3	pFT263	TadCBEa	multiplex array 01	60	26	25	8
4	pFT264	TadCBEd	multiplex array 01	60	23	21	5
5	pFT265	TadCBEd_V106W	multiplex array 01	60	55	18	16
6	pFT266	TadDE	multiplex array 01	60	45	2	2
7	pFT272	A3A_Y130F	multiplex array 02	60	50	30	13
8	pFT273	ABE8e	multiplex array 02	60	40	30	9
9	pFT274	TadCBEa	multiplex array 02	60	18	18	14
10	pFT275	TadCBEd	multiplex array 02	60	45	18	14
11	pFT276	TadCBEd_V106W	multiplex array 02	60	45	13	13
12	pFT277	TadDE	multiplex array 02	60	23	18	15

Supplementary Table 4. Base editing efficiency in regenerated rice plants.

Edit type	System	Transgenic T0 lines	target site	No. of mutation T0 lines	Ratio of mutation T0 lines	No. of biallelic T0 lines	Ratio of biallelic T0 lines
C to T	A3A_Y130F	3	OsAAT-sgRNA02	2	0.666666667	1	0.333333333
			OsACC-sgRNA02	2	0.666666667	2	0.666666667
			OsALS-sgRNA02	2	0.666666667	2	0.666666667
			OsCDC48-sgRNA02	2	0.666666667	0	0
			OsCGRS56-sgRNA01	1	0.333333333	1	0.333333333
			OsDEP1-sgRNA02	2	0.666666667	2	0.666666667
			OsGS3-sgRNA02	3	1	3	1
			OsLDMAR-sgRNA01	2	0.666666667	2	0.666666667
			OsPDS-sgRNA01	3	1	3	1
			OsSPL14-sgRNA01	3	1	1	0.333333333
C to T	ABE8e	7	OsAAT-sgRNA02	0	0	0	0
			OsACC-sgRNA02	0	0	0	0
			OsALS-sgRNA02	0	0	0	0
			OsCDC48-sgRNA02	0	0	0	0
			OsCGRS56-sgRNA01	0	0	0	0
			OsDEP1-sgRNA02	0	0	0	0
			OsGS3-sgRNA02	0	0	0	0
			OsLDMAR-sgRNA01	0	0	0	0
			OsPDS-sgRNA01	0	0	0	0
			OsSPL14-sgRNA01	0	0	0	0
C to T	TadCBEa	14	OsAAT-sgRNA02	14	1	14	1
			OsACC-sgRNA02	2	0.142857143	0	0
			OsALS-sgRNA02	11	0.785714286	8	0.571428571
			OsCDC48-sgRNA02	11	0.785714286	4	0.285714286
			OsCGRS56-sgRNA01	6	0.428571429	3	0.214285714
			OsDEP1-sgRNA02	8	0.571428571	4	0.285714286
			OsGS3-sgRNA02	12	0.857142857	4	0.285714286
			OsLDMAR-sgRNA01	13	0.928571429	12	0.857142857
			OsPDS-sgRNA01	13	0.928571429	12	0.857142857
			OsSPL14-sgRNA01	14	1	11	0.785714286
C to T	TadCBEd	10	OsAAT-sgRNA02	10	1	8	0.8
			OsACC-sgRNA02	8	0.8	7	0.7
			OsALS-sgRNA02	10	1	9	0.9
			OsCDC48-sgRNA02	10	1	8	0.8
			OsCGRS56-sgRNA01	8	0.8	6	0.6
			OsDEP1-sgRNA02	6	0.6	5	0.5
			OsGS3-sgRNA02	10	1	10	1
			OsLDMAR-sgRNA01	10	1	9	0.9
			OsPDS-sgRNA01	10	1	8	0.8
			OsSPL14-sgRNA01	10	1	8	0.8
C to T	TadCBEd_V106W	11	OsAAT-sgRNA02	10	0.909090909	8	0.727272727
			OsACC-sgRNA02	2	0.181818182	0	0
			OsALS-sgRNA02	7	0.636363636	4	0.363636364
			OsCDC48-sgRNA02	6	0.545454545	2	0.181818182
			OsCGRS56-sgRNA01	3	0.272727273	3	0.272727273
			OsDEP1-sgRNA02	6	0.545454545	3	0.272727273
			OsGS3-sgRNA02	8	0.727272727	6	0.545454545
			OsLDMAR-sgRNA01	8	0.727272727	5	0.454545455
			OsPDS-sgRNA01	9	0.818181818	8	0.727272727
			OsSPL14-sgRNA01	6	0.545454545	2	0.181818182
C to T	TadDE	12	OsAAT-sgRNA02	10	0.833333333	8	0.666666667
			OsACC-sgRNA02	1	0.083333333	0	0
			OsALS-sgRNA02	9	0.75	6	0.5
			OsCDC48-sgRNA02	7	0.583333333	5	0.416666667
			OsCGRS56-sgRNA01	9	0.75	6	0.5
			OsDEP1-sgRNA02	3	0.25	1	0.083333333

Edit type	System	Transgenic T0 lines	target site	No. of mutation T0 lines	Ratio of mutation T0 lines	No. of biallelic T0 lines	Ratio of biallelic T0 lines
			OsGS3-sgRNA02	6	0.5	3	0.25
			OsLDMAR-sgRNA01	9	0.75	8	0.666666667
			OsPDS-sgRNA01	11	0.916666667	9	0.75
			OsSPL14-sgRNA01	8	0.666666667	7	0.583333333
A to G	A3A_Y130F	3	OsAAT-sgRNA02	0	0	0	0
			OsACC-sgRNA02	0	0	0	0
			OsALS-sgRNA02	0	0	0	0
			OsCDC48-sgRNA02	0	0	0	0
			OsCGRS56-sgRNA01	0	0	0	0
			OsDEP1-sgRNA02	0	0	0	0
			OsGS3-sgRNA02	0	0	0	0
			OsLDMAR-sgRNA01	0	0	0	0
			OsPDS-sgRNA01	0	0	0	0
			OsSPL14-sgRNA01	0	0	0	0
A to G	ABE8e	7	OsAAT-sgRNA02	0	0	0	0
			OsACC-sgRNA02	3	0.428571429	3	0.428571429
			OsALS-sgRNA02	4	0.571428571	3	0.428571429
			OsCDC48-sgRNA02	2	0.285714286	1	0.142857143
			OsCGRS56-sgRNA01	3	0.428571429	2	0.285714286
			OsDEP1-sgRNA02	3	0.428571429	3	0.428571429
			OsGS3-sgRNA02	3	0.428571429	1	0.142857143
			OsLDMAR-sgRNA01	2	0.285714286	2	0.285714286
			OsPDS-sgRNA01	0	0	0	0
			OsSPL14-sgRNA01	0	0	0	0
A to G	TadCBEa	14	OsAAT-sgRNA02	0	0	0	0
			OsACC-sgRNA02	2	0.142857143	1	0.071428571
			OsALS-sgRNA02	3	0.214285714	0	0
			OsCDC48-sgRNA02	0	0	0	0
			OsCGRS56-sgRNA01	1	0.071428571	0	0
			OsDEP1-sgRNA02	2	0.142857143	0	0
			OsGS3-sgRNA02	0	0	0	0
			OsLDMAR-sgRNA01	4	0.285714286	0	0
			OsPDS-sgRNA01	0	0	0	0
			OsSPL14-sgRNA01	0	0	0	0
A to G	TadCBEd	10	OsAAT-sgRNA02	0	0	0	0
			OsACC-sgRNA02	0	0	0	0
			OsALS-sgRNA02	1	0.1	0	0
			OsCDC48-sgRNA02	0	0	0	0
			OsCGRS56-sgRNA01	0	0	0	0
			OsDEP1-sgRNA02	0	0	0	0
			OsGS3-sgRNA02	0	0	0	0
			OsLDMAR-sgRNA01	0	0	0	0
			OsPDS-sgRNA01	0	0	0	0
			OsSPL14-sgRNA01	0	0	0	0
A to G	TadCBEd_V106W	11	OsAAT-sgRNA02	0	0	0	0
			OsACC-sgRNA02	0	0	0	0
			OsALS-sgRNA02	0	0	0	0
			OsCDC48-sgRNA02	0	0	0	0
			OsCGRS56-sgRNA01	0	0	0	0
			OsDEP1-sgRNA02	1	0.090909091	0	0
			OsGS3-sgRNA02	0	0	0	0
			OsLDMAR-sgRNA01	0	0	0	0
			OsPDS-sgRNA01	0	0	0	0
			OsSPL14-sgRNA01	0	0	0	0
A to G	TadDE	12	OsAAT-sgRNA02	0	0	0	0
			OsACC-sgRNA02	0	0	0	0
			OsALS-sgRNA02	0	0	0	0
			OsCDC48-sgRNA02	0	0	0	0

Edit type	System	Transgenic T0 lines	target site	No. of mutation T0 lines	Ratio of mutation T0 lines	No. of biallelic T0 lines	Ratio of biallelic T0 lines
			OsCGRS56-sgRNA01	1	0.083333333	0	0
			OsDEP1-sgRNA02	0	0	0	0
			OsGS3-sgRNA02	0	0	0	0
			OsLDMAR-sgRNA01	0	0	0	0
			OsPDS-sgRNA01	0	0	0	0
			OsSPL14-sgRNA01	0	0	0	0

Supplementary Table 5. Potential sgRNA-dependent off-target sites and ratio of off-target events.

Treatment	sgRNA ID	Protospacer sequence_PAM	Potential off-target sites (protospacer_PAM)	Position	Mismatches	Test lines	Off-target lines	Ratio of off-target lines
Tissue	OsAAT-sgRNA02	CTGCGCCCCGCGCACGTTAG CGG	CTGCtCCCCGCGCACGTcAa AGG	chr2: 8267185	3	3	0	0.00%
	OsACC-sgRNA02	TCCACAGCTATCACACCCAC TGG	TCCACAGCTATgACACCaAC TGG	chr10: 10951932	2	3	0	0.00%
			TCCACcGCTATCtCACCCcC AGG	chr1: 14541328	3	3	0	0.00%
			TCCACgGCTATCtCACCCcC AGG	chr7: 9322540	3	3	0	0.00%
			TCCACtGCTATCtCACCCcC AGG	chr8: 24476471	3	3	0	0.00%
	OsALS-sgRNA02	TCTCCATGGACGCGCCGCCC GGG	TCTCCATcGACGCGCCGCCC GGG	chr4: 18956324	1	3	0	0.00%
			TCTCCATtGACGCGCCGCCC GGG	chr4: 18982110	1	3	0	0.00%
			cCTCCAcGcACGCGCCGCCC CGG	chr12: 410111	3	3	0	0.00%
			TCcCCtTGcACGCGCCGCCC TGG	chr6: 20921050	3	3	0	0.00%
	OsCDC48-sgRNA02	TAGCACCCATGACAATGACA TGG	none	none: none	none	3	none	none
	OsCGRS56-sgRNA01	CCCAACCCCCACTGACACCT AGG	gCCAACtCCCACTGACACCT GGG	chr12: 1670370	2	3	0	0.00%
			CCCctCtCCCACTGACACCT GGG	chr7: 26483710	3	3	0	0.00%
			CtCcACCCCCACTGACACaT GGG	chr2: 504258	3	3	0	0.00%
			CCCAACaCCCACaGACACgT GGG	chr6: 11076075	3	3	0	0.00%
	OsDEP1-sgRNA02	AGCACATGAGAGAACAATAT TGG	AGCACATGAGAGAACtcTtT TGG	chr10: 10910384	3	3	0	0.00%
			tGCACATGAGAGgACAAcAT GGG	chr3: 588931	3	3	0	0.00%
	OsGS3-sgRNA02	cTACGCGCATCTCTATATTT CGG	none	none: none	none	3	none	none
	OsLDMAR-sgRNA01	GTGCATCCTGGGAAAGCTTG TGG	none	none: none	none	3	none	none
	OsPDS-sgRNA01	GTTGGTCTTTGCTCCTGCAG AGG	GTTGtTCTcTGCaCCTGCAG GGG	chr2: 27877782	3	3	0	0.00%
	OsSPL14-sgRNA01	TCTTCTGTCAACCCAGCCAT GGG	TCTTCTGTCAACtCAGCCAT GGG	chr9: 18917895	1	3	0	0.00%
TadCBEa	OsAAT-sgRNA02	CTGCGCCCCGCGCACGTTAG CGG	CTGCtCCCCGCGCACGTcAa AGG	chr2: 8267185	3	14	0	0.00%
	OsACC-sgRNA02	TCCACAGCTATCACACCCAC TGG	TCCACAGCTATgACACCaAC TGG	chr10: 10951932	2	14	0	0.00%
			TCCACcGCTATCtCACCCcC AGG	chr1: 14541328	3	14	0	0.00%
			TCCACgGCTATCtCACCCcC AGG	chr7: 9322540	3	14	0	0.00%
			TCCACtGCTATCtCACCCcC AGG	chr8: 24476471	3	14	0	0.00%
	OsALS-sgRNA02	TCTCCATGGACGCGCCGCCC GGG	TCTCCATcGACGCGCCGCCC GGG	chr4: 18956324	1	14	7	50.00%
			TCTCCATtGACGCGCCGCCC GGG	chr4: 18982110	1	14	7	50.00%
			cCTCCAcGcACGCGCCGCCC CGG	chr12: 410111	3	14	2	14.29%
			TCcCCtTGcACGCGCCGCCC TGG	chr6: 20921050	3	14	0	0.00%
	OsCDC48-sgRNA02	TAGCACCCATGACAATGACA TGG	none	none: none	none	14	none	none
	OsCGRS56-sgRNA01	CCCAACCCCCACTGACACCT AGG	gCCAACtCCCACTGACACCT GGG	chr12: 1670370	2	14	0	0.00%
			CCCctCtCCCACTGACACCT GGG	chr7: 26483710	3	14	0	0.00%
			CtCcACCCCCACTGACACaT GGG	chr2: 504258	3	14	0	0.00%
			CCCAACaCCCACaGACACgT GGG	chr6: 11076075	3	14	0	0.00%
	OsDEP1-sgRNA02	AGCACATGAGAGAACAATAT TGG	AGCACATGAGAGAACtcTtT TGG	chr10: 10910384	3	14	0	0.00%
			tGCACATGAGAGgACAAcAT GGG	chr3: 588931	3	14	0	0.00%
	OsGS3-sgRNA02	cTACGCGCATCTCTATATTT CGG	none	none: none	none	14	none	none
	OsLDMAR-sgRNA01	GTGCATCCTGGGAAAGCTTG TGG	none	none: none	none	14	none	none
	OsPDS-sgRNA01	GTTGGTCTTTGCTCCTGCAG AGG	GTTGtTCTcTGCaCCTGCAG GGG	chr2: 27877782	3	14	0	0.00%
	OsSPL14-sgRNA01	TCTTCTGTCAACCCAGCCAT GGG	TCTTCTGTCAACtCAGCCAT GGG	chr9: 18917895	1	14	9	64.29%
TadDE	OsAAT-sgRNA02	CTGCGCCCCGCGCACGTTAG CGG	CTGCtCCCCGCGCACGTcAa AGG	chr2: 8267185	3	12	0	0.00%
	OsACC-sgRNA02	TCCACAGCTATCACACCCAC TGG	TCCACAGCTATgACACCaAC TGG	chr10: 10951932	2	12	0	0.00%
			TCCACcGCTATCtCACCCcC AGG	chr1: 14541328	3	12	0	0.00%
			TCCACgGCTATCtCACCCcC AGG	chr7: 9322540	3	12	0	0.00%
			TCCACtGCTATCtCACCCcC AGG	chr8: 24476471	3	12	0	0.00%
	OsALS-sgRNA02	TCTCCATGGACGCGCCGCCC GGG	TCTCCATcGACGCGCCGCCC GGG	chr4: 18956324	1	12	4	33.33%

Treatment	sgRNA ID	Protospacer sequence_PAM	Potential off-target sites (protospacer_PAM)	Position	Mismatches	Test lines	Off-target lines	Ratio of off-target lines
			TCTCCATtGACGCGCCGCCC GGG	chr4: 18982110	1	12	8	66.67%
			cCTCCAcGcACGCGCCGCCC CGG	chr12: 410111	3	12	0	0.00%
			TCcCCtTGcACGCGCCGCCC TGG	chr6: 20921050	3	12	0	0.00%
	OsCDC48-sgRNA02	TAGCACCCATGACAATGACA TGG	none	none: none	none	12	none	none
	OsCGRS56-sgRNA01	CCCAACCCCCACTGACACCT AGG	gCCAACtCCCACTGACACCT GGG	chr12: 1670370	2	12	0	0.00%
			CCCctCtCCCACTGACACCT GGG	chr7: 26483710	3	12	0	0.00%
			CtCcACCCCCACTGACACaT GGG	chr2: 504258	3	12	0	0.00%
			CCCAACaCCCACaGACACgT GGG	chr6: 11076075	3	12	0	0.00%
	OsDEP1-sgRNA02	AGCACATGAGAGAACAATAT TGG	AGCACATGAGAGAACtcTtT TGG	chr10: 10910384	3	12	0	0.00%
			tGCACATGAGAGgACAAcAT GGG	chr3: 588931	3	12	0	0.00%
	OsGS3-sgRNA02	cTACGCGCATCTCTATATTT CGG	none	none: none	none	12	none	none
	OsLDMAR-sgRNA01	GTGCATCCTGGGAAAGCTTG TGG	none	none: none	none	12	none	none
	OsPDS-sgRNA01	GTTGGTCTTTGCTCCTGCAG AGG	GTTGtTCTcTGCaCCTGCAG GGG	chr2: 27877782	3	12	0	0.00%
	OsSPL14-sgRNA01	TCTTCTGTCAACCCAGCCAT GGG	TCTTCTGTCAACtCAGCCAT GGG	chr9: 18917895	1	12	3	25.00%

Supplementary Table 6. Detailed information on WGS sequencing depth.

Group	ID	Sequencing depth (X)	mapping ratio (%)	Genome coverage (%)
TadCBEa	TadCBEa_02	32.31	98.74	99.35
TadCBEa	TadCBEa_03	32.88	98.63	99.21
TadCBEa	TadCBEa_05	34	98.58	99.38
TadCBEa	TadCBEa_07	33.58	98.52	99.39
TadCBEa	TadCBEa_08	34.47	98.53	99.46
TadCBEa	TadCBEa_09	31.44	98.63	99.41
TadCBEa	TadCBEa_10	32.58	98.66	99.48
TadCBEa	TadCBEa_11	33.57	98.47	99.48
TadCBEa	TadCBEa_12	31.87	98.69	99.45
TadCBEa	TadCBEa_13	30.72	98.7	98.94
TadCBEa	TadCBEa_14	28.7	98.06	99.12
TadCBEa	TadCBEa_15	28.58	98.87	99.23
TadCBEa	TadCBEa_17	29.11	98.69	99.25
TadCBEa	TadCBEa_18	28.46	98.56	99.3
TadDE	TadDE_02	31.7	98.62	99.56
TadDE	TadDE_03	33.19	98.3	99.56
TadDE	TadDE_04	33.27	98.4	99.62
TadDE	TadDE_06	34.48	98.52	99.61
TadDE	TadDE_07	32.35	98.32	99.57
TadDE	TadDE_09	34.31	98.42	99.62
TadDE	TadDE_10	34.45	98.54	99.6
TadDE	TadDE_11	33.17	98.57	99.6
TadDE	TadDE_12	32.67	98.38	99.49
TadDE	TadDE_13	28.86	98.21	99.35
TadDE	TadDE_14	30.18	98.85	99.22
TadDE	TadDE_15	30.88	98.75	99.07
Tissue	Tissue_01	39.81	98.04	99.18
Tissue	Tissue_02	33.71	98.06	99.23
Tissue	Tissue_03	41	98.12	99.37

Supplementary Table 7. Detailed information on RNA-seq analysis.

Group	ID	Total mapped reads (M)
TadCBEa	TadCBEa_02	47.72
TadCBEa	TadCBEa_03	43.77
TadCBEa	TadCBEa_05	42.67
TadCBEa	TadCBEa_07	42.9
TadCBEa	TadCBEa_08	42.51
TadDE	TadDE_02	42.44
TadDE	TadDE_03	41.39
TadDE	TadDE_04	42.61
TadDE	TadDE_11	42.65
Tissue	Tissue_01	41.35
Tissue	Tissue_02	43.79
Tissue	Tissue_03	44.26
