# Supplementary material for: High performance TadA-8e derived cytosine and dual base editors with undetectable off-target effects in plants
Source: Nat Commun. 2024 Jun 14;15:5103. doi: 10.1038/s41467-024-49473-w (PMC11178825; doi:10.1038/s41467-024-49473-w)
Supplement: Supplementary file 3 — Description of Additional Supplementary Files [file 41467_2024_49473_MOESM3_ESM.pdf]

Description of Additional Supplementary Files

File Name: Supplementary Data 1

Description: Oligos and gBlocks used in this study.
