# Supplementary material for: High performance TadA-8e derived cytosine and dual base editors with undetectable off-target effects in plants
Source: Nat Commun. 2024 Jun 14;15:5103. doi: 10.1038/s41467-024-49473-w (PMC11178825; doi:10.1038/s41467-024-49473-w)
Supplement: Supplementary file 5 — Reporting Summary [file 41467_2024_49473_MOESM5_ESM.pdf]

nature portfolio

Corresponding author(s): Yong Zhang, Yiping Qi and Tao Zhang

Last updated by author(s): May 20, 2024

Reporting Summary

Nature Portfolio wishes to improve the reproducibility of the work that we publish. This form provides structure for consistency and transparency in reporting. For further information on Nature Portfolio policies, see our Editorial Policies and the Editorial Policy Checklist.

Statistics

For all statistical analyses, confirm that the following items are present in the figure legend, table legend, main text, or Methods section.

n/a	Confirmed	
☐	☒	The exact sample size (*n*) for each experimental group/condition, given as a discrete number and unit of measurement
☐	☒	A statement on whether measurements were taken from distinct samples or whether the same sample was measured repeatedly
☐	☒	The statistical test(s) used AND whether they are one- or two-sided<br>*Only common tests should be described solely by name; describe more complex techniques in the Methods section.*
☒	☐	A description of all covariates tested
☒	☐	A description of any assumptions or corrections, such as tests of normality and adjustment for multiple comparisons
☐	☒	A full description of the statistical parameters including central tendency (e.g. means) or other basic estimates (e.g. regression coefficient) AND variation (e.g. standard deviation) or associated estimates of uncertainty (e.g. confidence intervals)
☐	☒	For null hypothesis testing, the test statistic (e.g. *F*, *t*, *r*) with confidence intervals, effect sizes, degrees of freedom and *P* value noted<br>*Give P values as exact values whenever suitable.*
☒	☐	For Bayesian analysis, information on the choice of priors and Markov chain Monte Carlo settings
☒	☐	For hierarchical and complex designs, identification of the appropriate level for tests and full reporting of outcomes
☒	☐	Estimates of effect sizes (e.g. Cohen's *d*, Pearson's *r*), indicating how they were calculated

Our web collection on statistics for biologists contains articles on many of the points above.

Software and code

Policy information about availability of computer code

Data collection	No software were used for data collection. Public datasets were collected from NCBI, The Rice Annotation Project Database (RAP-DB), Rice Genome Annotation Project (MSU). Detailed information are available in Methods.
Data analysis	Data analysis was performed using the following software: CRIPSRMatch (https://github.com/zhangtaolab/CRISPRMatch), GraphPad Prism 9.4.1. WGS data analysis was conducted using the SKEWER_V0.2.2, BWA mem tool_V0.7.17, Picard tools and Samtools_V1.9, GATK_V3.8, LoFreq_V2.1.2, Mutect2, VarScan2_V2.4.3, Pindel_V0.2, Bedtools_V2.27.1, Cas-OFFinder_V2.4, and Circos_V0.69. RNA-seq data analysis was conducted using Hisat2_V2.2.0, GATK_V3.8, VariantFiltration tool, Python, R, and WebLogo3 (http://weblogo.threeplusone.com/).

For manuscripts utilizing custom algorithms or software that are central to the research but not yet described in published literature, software must be made available to editors and reviewers. We strongly encourage code deposition in a community repository (e.g. GitHub). See the Nature Portfolio guidelines for submitting code & software for further information.

Data

Policy information about availability of data

All manuscripts must include a data availability statement. This statement should provide the following information, where applicable:

- Accession codes, unique identifiers, or web links for publicly available datasets
- A description of any restrictions on data availability
- For clinical datasets or third party data, please ensure that the statement adheres to our policy

Key vectors used in this study have been deposited to Addgene: pGEL850 (expression TadCBEa in rice cells, Addgene # 214250); pGEL851 (expression TadCBEd in rice cells, Addgene # 214251); pGEL852 (expression TadCBEd_V106W in rice cells, Addgene # 214252); pGEL853 (expression TadDE in rice cells, Addgene # 214253); pGEL854 (expression eTd-CBE in rice cells, Addgene # 214254); pGEL855 (expression Td_CBEmax in rice cells, Addgene # 214255); pGEL856 (expression TadDE-NG in rice cells, Addgene # 214256); pYPQ265E6 (TadCBEa-nzCas9-2xUGI, Addgene # 213469); pYPQ265E7 (TadCBEd-nzCas9-2xUGI, Addgene # 213470); pYPQ265E8 (TadCBEd-V106W-nzCas9-2xUGI, Addgene # 213471); pYPQ265E9 (TadDE-nzCas9-2xUGI, Addgene # 213472). The plasmids mentioned above have been submitted to the Addgene (https://www.addgene.org/browse/article/28243932/). The NGS data generated in this study have been deposited in the National Center for Biotechnology Information (NCBI) database under Sequence Read Archive (SRA) with the BioProject ID PRJNA1054338 (https://dataview.ncbi.nlm.nih.gov/object/PRJNA1054338). Similarly, the WGS and RNA-seq data have been deposited in the NCBI database under SRA with the BioProject ID PRJNA1059495 (https://dataview.ncbi.nlm.nih.gov/object/PRJNA1059495). Source data are provided with this paper.

Research involving human participants, their data, or biological material

Policy information about studies with human participants or human data. See also policy information about sex, gender (identity/presentation), and sexual orientation and race, ethnicity and racism.

Reporting on sex and gender	N/A
Reporting on race, ethnicity, or other socially relevant groupings	N/A
Population characteristics	N/A
Recruitment	N/A
Ethics oversight	N/A

Note that full information on the approval of the study protocol must also be provided in the manuscript.

Field-specific reporting

Please select the one below that is the best fit for your research. If you are not sure, read the appropriate sections before making your selection.

☒ Life sciences ☐ Behavioural & social sciences ☐ Ecological, evolutionary & environmental sciences

For a reference copy of the document with all sections, see nature.com/documents/nr-reporting-summary-flat.pdf

Life sciences study design

All studies must disclose on these points even when the disclosure is negative.

Sample size	The sample size for our current study was determined based on the specific requirement of each experiment to have sufficient statistical power, as well as accepted sample sizes in relevant published reports within the field (Song et al., 2022, Nat Biotechnol; Schindele,et al., 2022, Nat Commun; Zhou et al., 2023, Nat Plants) . The actual sample size for each experiment was fully described in either the figure legends or the Methods section.
Data exclusions	No data was excluded.
Replication	For the rice and tomato protoplast assays, three biological replicates were used for each construct. For the rice phenotypic assays, a minimum of three biological replicates were used for stable transgenic plants. True biological replicates (ie., independent plants) were used as replicates for statistical analyses. The actual number of replicates is stated in the Figure legends.
Randomization	All samples were randomly distributed across all replicates.
Blinding	For each experiment, the treatments were compared to a control treatment without any prior knowledge of whether the experimental variables being altered would have a positive or negative impact on the results. Moreover, no additional blinding was implemented.

Reporting for specific materials, systems and methods

We require information from authors about some types of materials, experimental systems and methods used in many studies. Here, indicate whether each material, system or method listed is relevant to your study. If you are not sure if a list item applies to your research, read the appropriate section before selecting a response.

Materials & experimental systems

n/a	Involved in the study	
☒	☐	Antibodies
☒	☐	Eukaryotic cell lines
☒	☐	Palaeontology and archaeology
☒	☐	Animals and other organisms
☒	☐	Clinical data
☒	☐	Dual use research of concern
☐	☒	Plants

Methods

n/a	Involved in the study	
☒	☐	ChIP-seq
☒	☐	Flow cytometry
☒	☐	MRI-based neuroimaging

Dual use research of concern

Policy information about dual use research of concern

Hazards

Could the accidental, deliberate or reckless misuse of agents or technologies generated in the work, or the application of information presented in the manuscript, pose a threat to:

No	Yes	
☒	☐	Public health
☒	☐	National security
☒	☐	Crops and/or livestock
☒	☐	Ecosystems
☒	☐	Any other significant area

Experiments of concern

Does the work involve any of these experiments of concern:

No	Yes	
☒	☐	Demonstrate how to render a vaccine ineffective
☒	☐	Confer resistance to therapeutically useful antibiotics or antiviral agents
☒	☐	Enhance the virulence of a pathogen or render a nonpathogen virulent
☒	☐	Increase transmissibility of a pathogen
☒	☐	Alter the host range of a pathogen
☒	☐	Enable evasion of diagnostic/detection modalities
☒	☐	Enable the weaponization of a biological agent or toxin
☒	☐	Any other potentially harmful combination of experiments and agents

Plants

Seed stocks	The Japonica cultivar Nipponbare of rice (Oryza sativa) and the tomato (Solanum lycopersicum) cultivar M82 were used in this study.
Novel plant genotypes	All the rice base editing mutants were generated using Agrobacterium-mediated rice transformation, employing both cytosine and dual base editors derived from TadA-8e, following the detailed protocol described in the manuscript.
Authentication	T-DNA insertion mutants were verified through PCR product Sanger sequencing, to preliminarily confirm the outcomes of base editing in rice plants. Subsequently, 10 to 15 lines of each base editing construct were subjected to whole-genome sequencing.
